# Supplementary material for: Transparent dynamic infrared emissivity regulators
Source: Nat Commun. 2023 Aug 22;14:5087. doi: 10.1038/s41467-023-40902-w (PMC10444874; doi:10.1038/s41467-023-40902-w)
Supplement: Supplementary file 1 — Supplementary Information [file 41467_2023_40902_MOESM1_ESM.pdf]

Supporting information for:

Transparent dynamic infrared emissivity regulators

Yan Jia[1], Dongqing Liu[1]*, Desui Chen[2], Yizheng Jin[2], Chen Chen[1], Jundong Tao[1], Haifeng Cheng[1]*, Shen Zhou[1, 3], Baizhang Cheng[1], Xinfei Wang[1], Zhen Meng[1], Tianwen Liu[1]

[1]Science and Technology on Advanced Ceramic Fibers and Composites Laboratory, College of Aerospace Science and Engineering, National University of Defense Technology, Changsha, P. R. China.

[2]Key Laboratory of Excited-State Materials of Zhejiang Province, State Key Laboratory of Silicon Materials, Department of Chemistry, Zhejiang University, Hangzhou, P. R. China.

[3]Institute for Quantum Science and Technology, College of Science, National University of Defense Technology, Changsha, P. R. China.

*Corresponding author. Email: liudongqing07@nudt.edu.cn (D. L.); hfcheng@rocketmail.com (H. C.)

Movie captions:

Movie S1: Real-time visible and infrared thermal movie of TDIE regulators, recorded by a Nikon D3100 digital camera and FLIR T1050sc infrared camera.

Movie S2: Real-time visible and infrared thermal movie of SES roofs and SES windows, showing different mode in Supplementary Fig. 41 and Fig. 42.

Movie S3: Real time visible and infrared thermal movie of multispectral display.

Supplementary Note 1. Calculation of emissivity and infrared radiation power.

Since the infrared transmittance of the TDIE regulator is 0,

$$\alpha = 1 - R \tag{S1}$$

where α and R are the infrared absorptivity and infrared reflectance of the TDIE regulator, respectively.

According to Kirchhoff's law,

$$\alpha_\lambda = \varepsilon_\lambda \tag{S2}$$

where ε_λ is the emissivity at wavelength λ and α_λ is the absorptivity at λ.

According to Eq. (S1) and (S2), the emissivity of the TDIE regulator (ε_λ) at λ can be calculated as follows:

$$\varepsilon_\lambda = 1 - R_\lambda \tag{S3}$$

The band emissivity of the TDIE regulators was calculated by integrating the total spectral emissivity over the blackbody radiation spectral range:

$$\varepsilon(\lambda_1, \lambda_2) = \frac{\int_{\lambda_1}^{\lambda_2} I_{BB}(T,\lambda)\varepsilon(T,\lambda)d\lambda}{\int_{\lambda_1}^{\lambda_2} I_{BB}(T,\lambda)d\lambda} \tag{S4}$$

We calculated (λ_1 and, λ_2) as bandwidths. I_{BB} (T, λ) is the spectral intensity emitted by the blackbody at λ and temperature T. Here, (λ_1, λ_2) refers to MWIR (3–5 μm) and LWIR (7.5–13 μm) in an infrared atmospheric window, respectively.

The infrared radiation power of the TDIE regulator in an infrared atmospheric window at T can be calculated as

$$P(\lambda_1, \lambda_2) = \varepsilon(\lambda_1, \lambda_2) \times \int_{\lambda_1}^{\lambda_2} I_{BB}(T, \lambda)d\lambda \tag{S5}$$

The change in the infrared radiation power (ΔP) of the TDIE regulator in different

emissivity states was computed as

$$\Delta P(\lambda_1, \lambda_2) = P_h(\lambda_1, \lambda_2) - P_l(\lambda_1, \lambda_2) \quad \text{(S6)}$$

where $P_h(\lambda_1, \lambda_2)$ and $P_l(\lambda_1, \lambda_2)$ represent the infrared radiation power of the TDIE regulator in the high-and low-emissivity states, respectively.

Supplementary Note 2. Constituent layer of the TDIE regulator.

The morphology of AZO NCs is shown in Supplementary Fig. 1(a, b). AZO NCs have a size of 11 ± 0.6 nm and a doping content of 0.95% (as measured by ICP-OES). The LSPR peak of AZO NCs with a peak position of 7.58 μm was shown in Supplementary Fig. 2. The relationship between the LSPR frequency (ω_{LSPR}) and free carrier concentration (n) of an NC is given as[1,2]:

$$\omega_{LSPR} = \sqrt{\frac{\omega_p^2}{1+2\varepsilon_m} - \gamma^2} \quad \text{(S7)}$$

$$\omega_p = \sqrt{\frac{ne^2}{\varepsilon_0 m_e}} \quad \text{(S8)}$$

where ω_P is the bulk plasma frequency of free carriers, γ is bulk collision frequency, ε_m is the environment dielectric constant, ε_0 is free space permittivity, and m_e is electron effective mass. The peak of LSPR was red-shifted to LWIR when compared to 13.1% Sn-doping ITO and 3.7% Al-doping AZO[3,4]. Furthermore, the long LSPR peak tail resulted in LSPR regulation across the entire MWIR and LWIR infrared atmospheric window range. The AZO NCs are tightly stacked to form the film with a thickness of ~1.14 μm (Supplementary Fig. 3). The evaporated ITO layer has a thickness of ~330 nm (Supplementary Fig. 3) and serves as the working electrode (7.56 Ω sq^{-1}) as well as high infrared reflection layer ($R_{3\text{-}13\ \mu m}$ = 86.9%). The visible transmittance of each constituent layer is shown in Supplementary Fig. 4. The visible light transmittance of BaF_2/AZO NCs film/ITO film half-device and AZO NCs film/ITO glass half-device is greater than 80%. Furthermore, the transparent liquid electrolyte increases the TDIE regulator's visible light transmittance to 84.7%. As shown in Supplementary Fig. 7, the

infrared emissivity of the TDIE regulator can change at ±2.5 V while maintaining the device's high visible transparency.

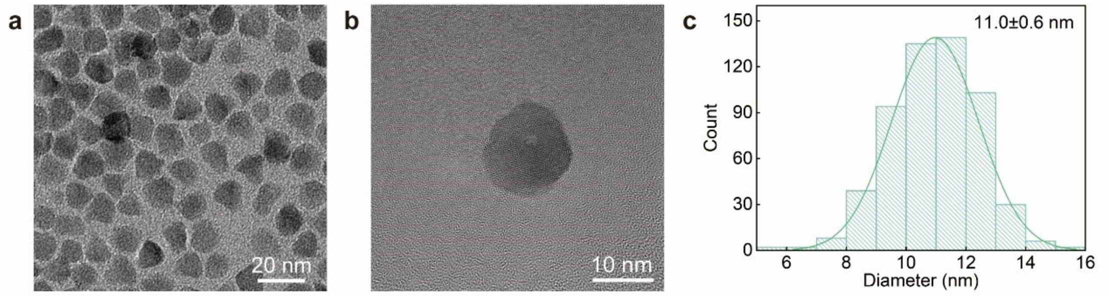

Supplementary Fig. 1 TEM image and size distributions of AZO NCs.

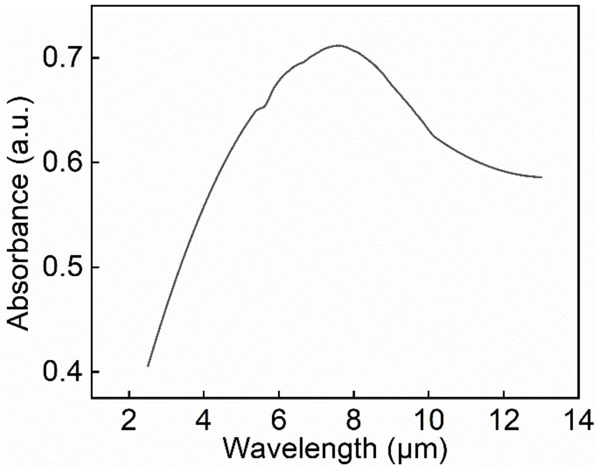

Supplementary Fig. 2 LSPR peak of AZO NCs showing a smooth absorption peak (7.58 μm).

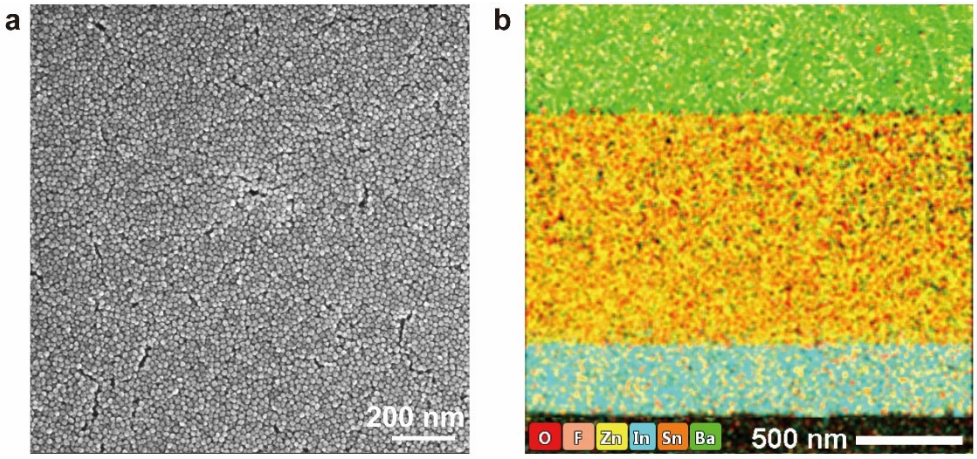

Supplementary Fig. 3 a, Surface morphology of an AZO NC film. **b,** Elemental map of BaF_2/AZO NC film/ITO film half-device.

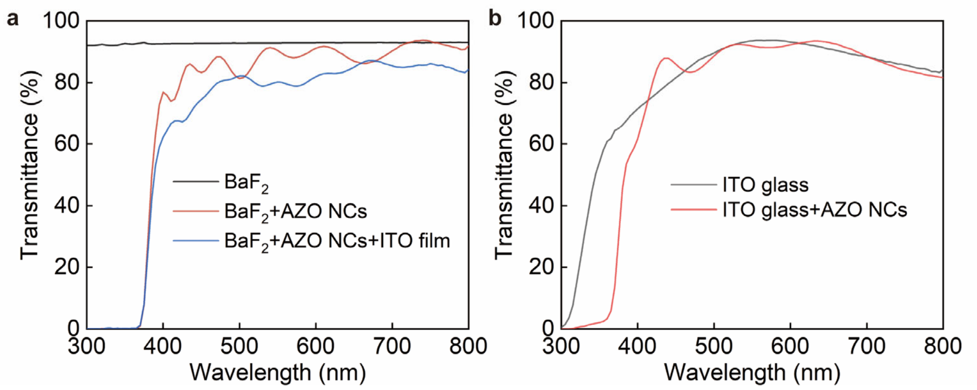

Supplementary Fig. 4 a, Visible light transmittance of BaF_2/AZO NC film/ITO film half-device. **b,** Visible light transmittance of AZO NC film/ITO glass half-device.

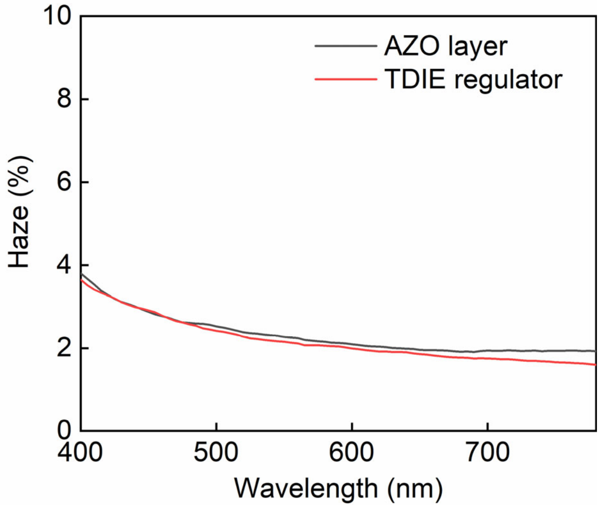

Supplementary Fig. 5 The haze of AZO film and TDIE regulator.

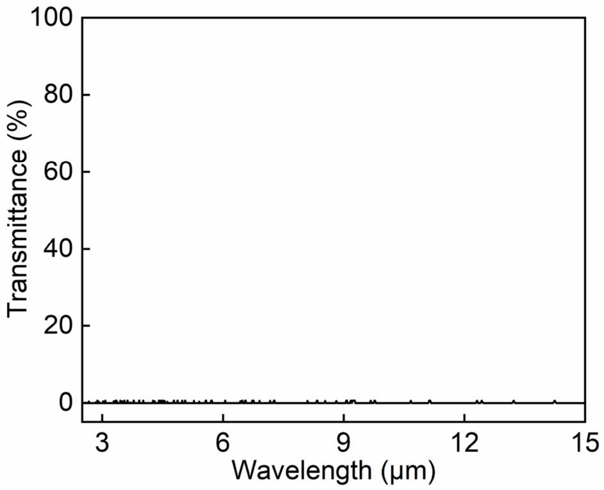

Supplementary Fig. 6 The infrared transmittance curve (2.5-15 μm) of the TDIE regulator.

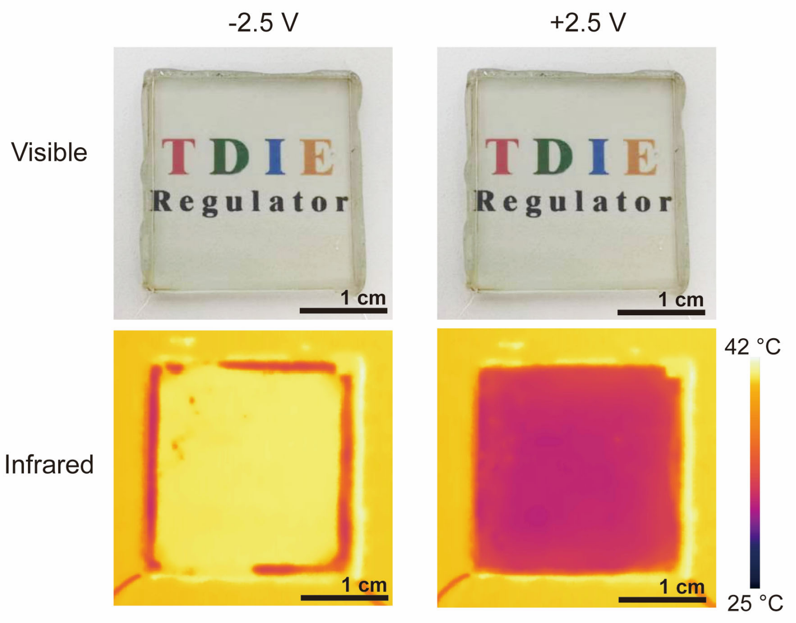

Supplementary Fig. 7 Photographs and LWIR images of TDIE regulator after applying ±2.5V voltage.

Supplementary Note 3. The properties of TDIE regulator.

Supplementary Fig. 9 shows the emissivity of TDIE regulators at different voltage. The emissivity at MWIR (3-5 μm) and LWIR (7.5-13 μm) at different voltage was calculated by Supplementary Equation S4. The maximum emissivity regulation of 0.51 at MWIR was calculated by the emissivity of TDIE regulators at -2.5 V and 2.5 V. The maximum emissivity regulation of 0.41 at LWIR was calculated by the emissivity of TDIE regulators at -2.5 V and 1 V.

The response time of the TDIE regulator began to decrease to 1-10 s after 4000 cycles, as shown in Supplementary Fig. 12, and then remains within ~10 s. As the number of cycles increases, the response time will slight increase. This is mainly due to the corrosion of the working ITO electrode after 4000 cycles. In addition, after multiple cycles, the adsorption/desorption rates of Li^+ in the electrolyte decrease on the working electrode and counter electrode. To reduce response time after multiple cycles, a promising avenue is the selection of an electrolyte with greater stability, reduced corrosiveness, and faster ion adsorption/desorption rates. The device still shows a fast response time after 10000 cycles compared with other infrared modulation devices[5-11].

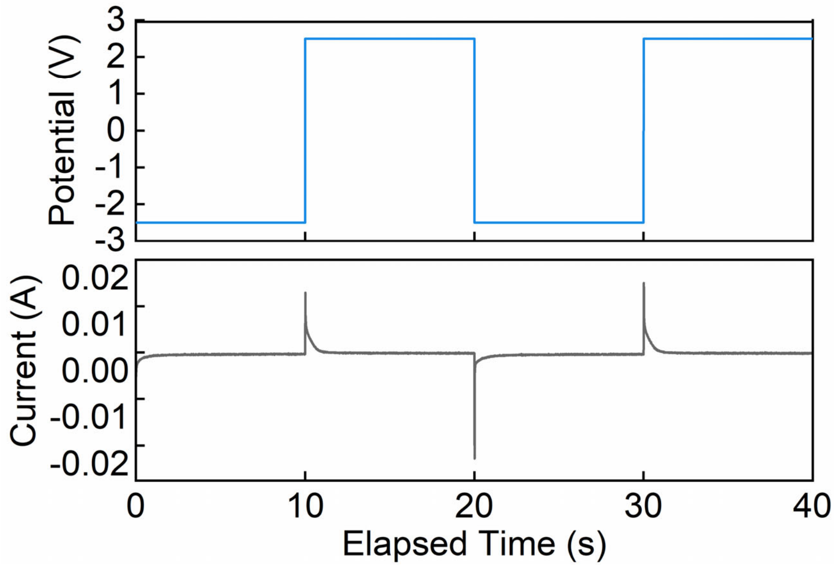

Supplementary Fig. 8 Current time response of a TDIE regulator under voltage cycling between ±2.5 V. Electrons are injected into the AZO film at 0.27 ± 0.5 mC/cm^2. The charging and discharging driving powers are 5.87 J/m^2 and 7.87 J/m^2, respectively.

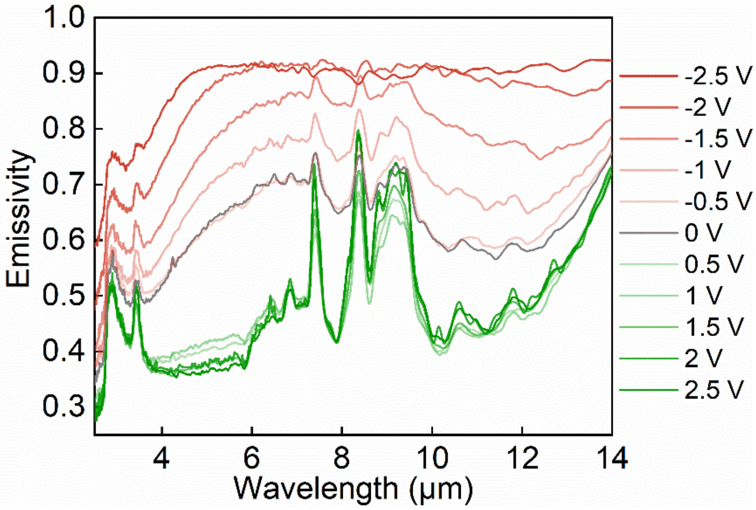

Supplementary Fig. 9 Emissivity of a TDIE regulator under applied voltages. The infrared spectrum was measured after 10 min of voltage application.

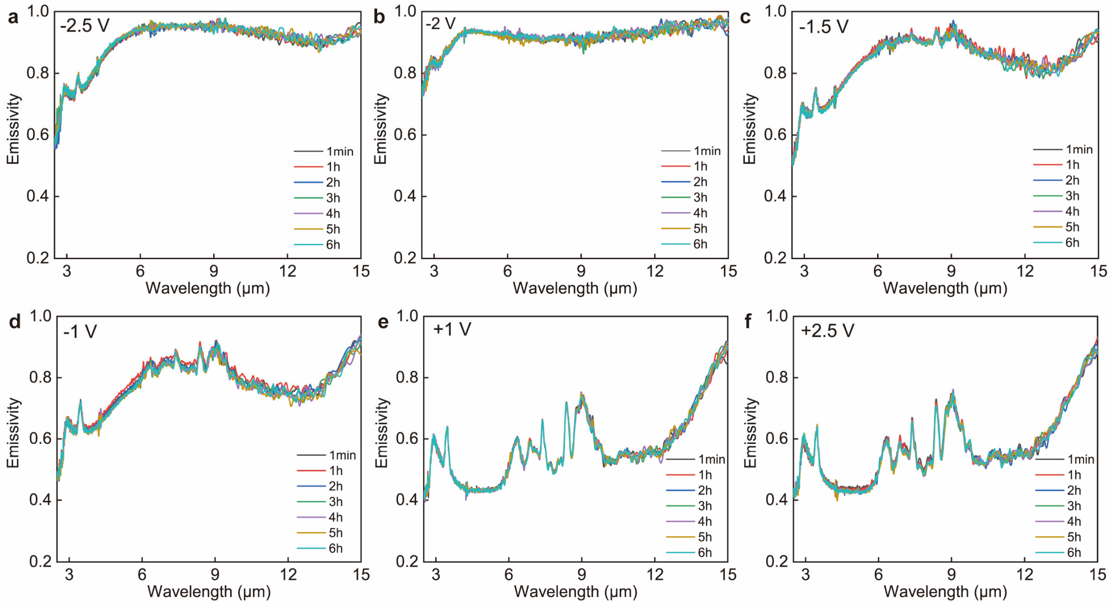

Supplementary Fig. 10 The emissivity of the TDIE regulator applied voltage of (**a**) -2.5 V, (**b**) -2 V, (**c**) -1.5 V, (**d**) -1 V, (**e**) +1 V, (**f**) +2.5 V over an extended time.

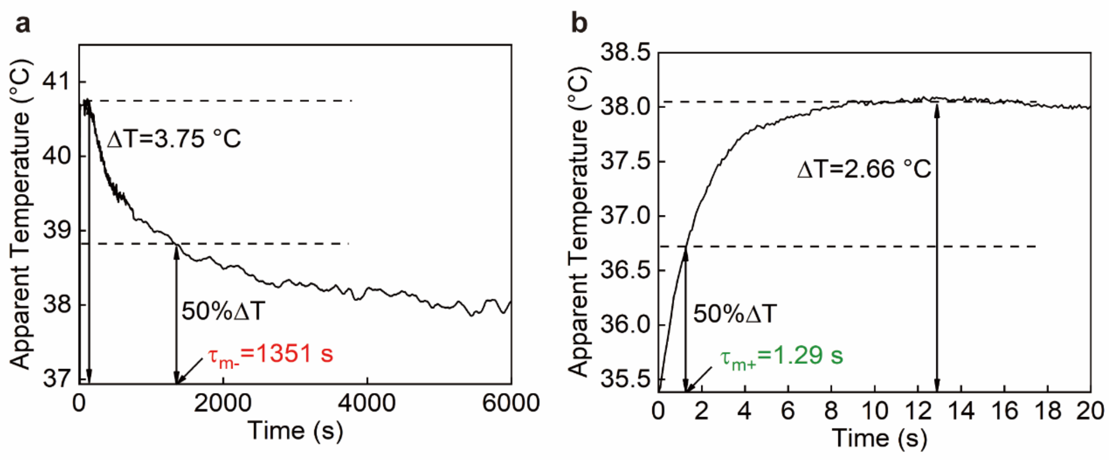

Supplementary Fig. 11 Memory time of a TDIE regulator after applying (**a**) -2.5 V or (**b**) 2.5 V. Memory time is the time required for the apparent temperature of the device to return to 50% of its initial temperature after the voltage is disconnected.

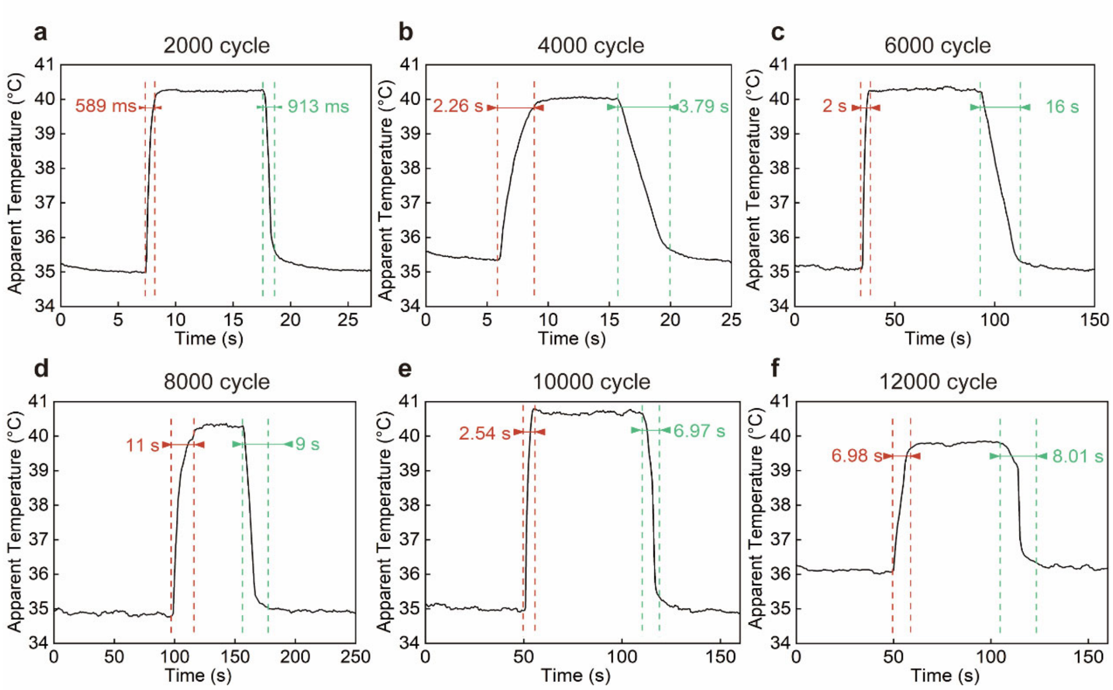

Supplementary Fig. 12 Response time of a TDIE regulator after (**a**) 2000, (**b**) 4000, (**c**) 6000, (**d**) 8000, (**e**) 10,000, and (**f**) 12,000 cycles.

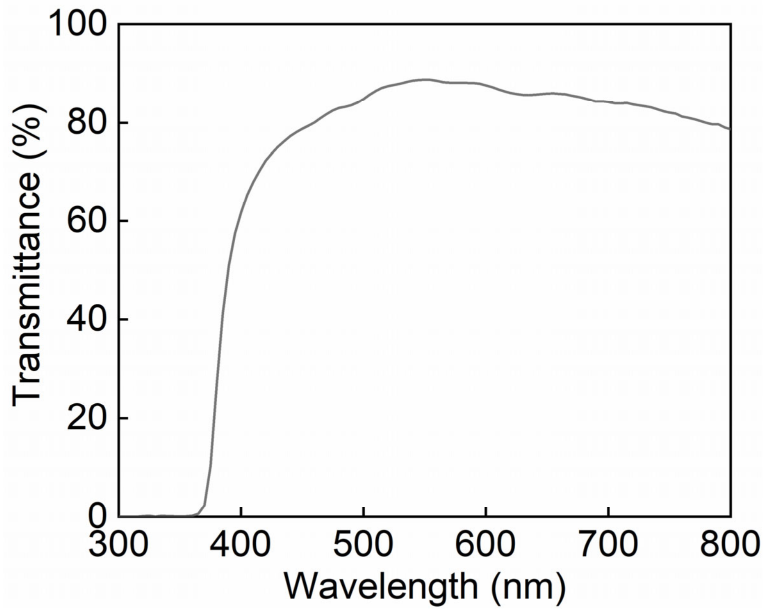

Supplementary Fig. 13 Visible light transmittance of a TDIE regulator after 10,000 cycles.

Supplementary Note 4. Long-term outdoor testing of TDIE regulator.

We set up an experimental device outdoors, shown in supplementary Fig. 14. We simulated the long-term operation of the TDIE regulator outdoors. The infrared thermal imager was used to record the apparent temperature of TDIE regulator under positive/negative operating voltages. TDIE regulator can operate stably under outdoor environment for over 6 days, shown in supplementary Fig. 18. During long-term operation of TDIE regulator, its visible light transmittance decreased to 58.9% (supplementary Fig. 19). This is because after applying voltage for a long time, Li^+ in the LiTFSI based electrolyte is reduced to metal Li and reacts with O_2, N_2, and water vapor in the air to form Li_2O, Li_3N, and LiOH. The contact between air and electrolyte may be due to the transparent silicone used in device encapsulation having a certain gas permeability. In addition, the AZO NC film also partially corroded and peeled off after long-term operation for more than 7 days (supplementary Fig. 20), which was due to the corrosion of the LiTFSI electrolyte.

We simulated the effect of dust covering on the TDIE regulator's infrared emissivity regulation performance (Supplementary Fig. 21). Dust is a high emissivity material. When a thin layer of dust covers the surface of the TDIE regulator and a voltage of +2.5V is applied, the dust covered area exhibits high emissivity due to the presence of dust while the dust uncovered area shows low emissivity characteristic of the TDIE regulator (Supplementary Fig. 21 (b)). When a thick layer of dust accumulated on the surface of the TDIE regulator, regardless of whether a positive or

negative voltage is applied, TDIE regulator exhibited high emissivity state of dust (Supplementary Fig. 21 (c)). On rainy days, raindrops will fall on the TDIE regulator (Supplementary Fig. 22). Since water is an infrared-opaque high-emissivity state, when the water film is covered on the TDIE regulator, the TDIE regulator will exhibit the high emissivity of the water film (Supplementary Fig. 22).

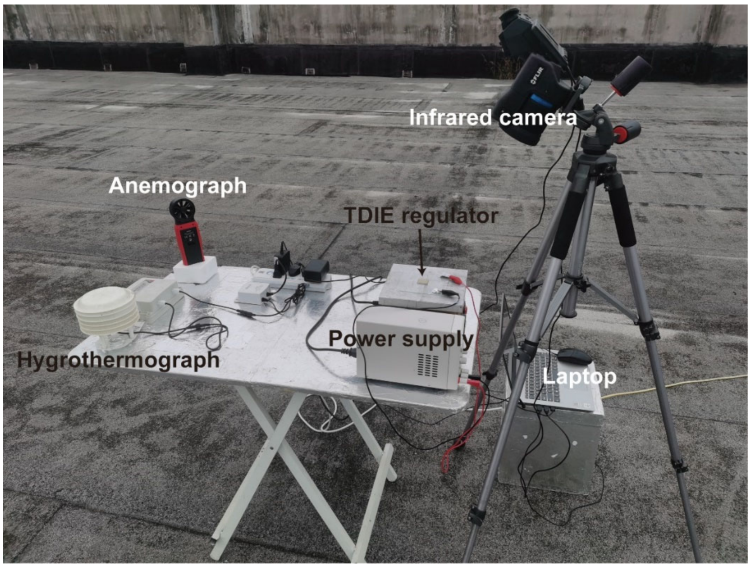

Supplementary Fig. 14 The test setup for TDIE regulator in outdoor environments.

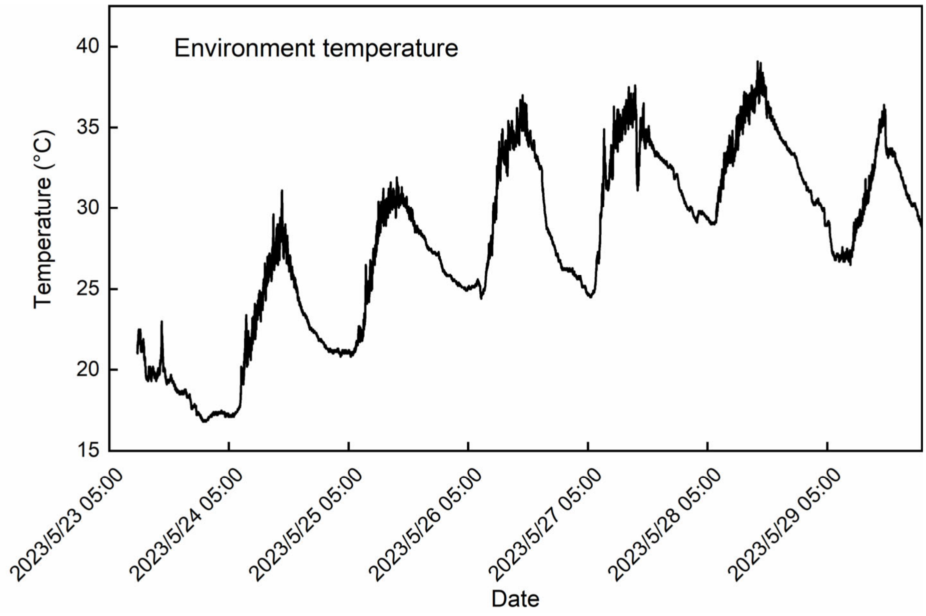

Supplementary Fig. 15 Environment temperature measured by

hygrothermograph (Date: 2023.5.23 to 2023.5.29; Location: Changsha, China).

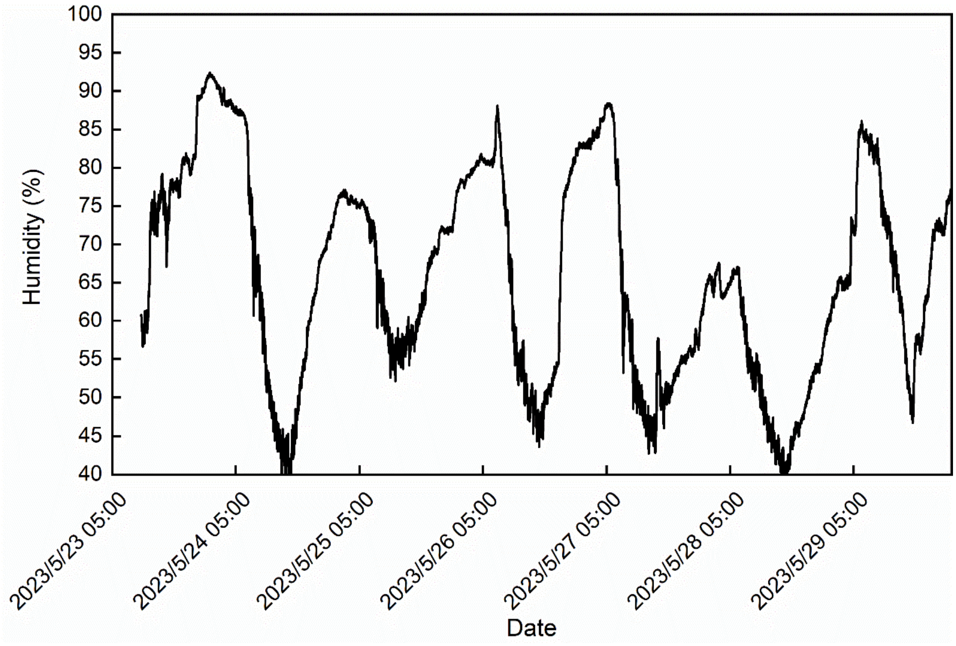

Supplementary Fig. 16 Humidity measured by hygrothermograph (Date: 2023.5.23 to 2023.5.29; Location: Changsha, China).

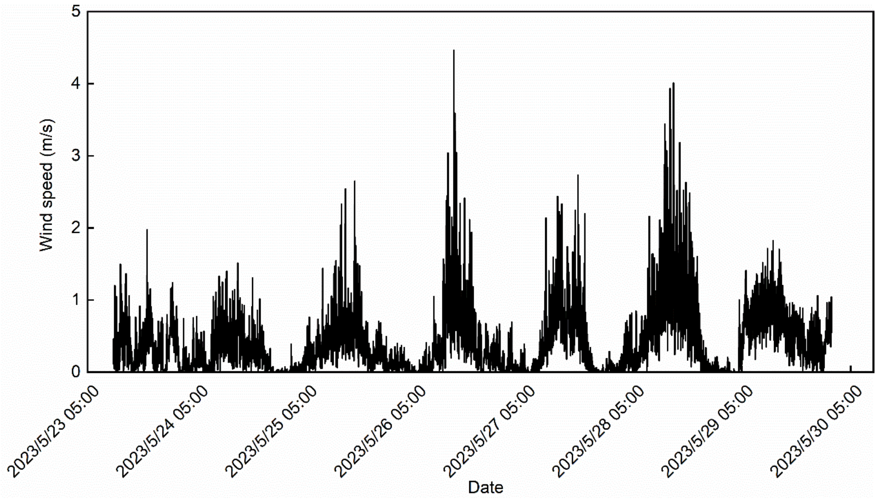

Supplementary Fig. 17 Wind speed measured by anemograph (Date: 2023.5.23 to 2023.5.29; Location: Changsha, China).

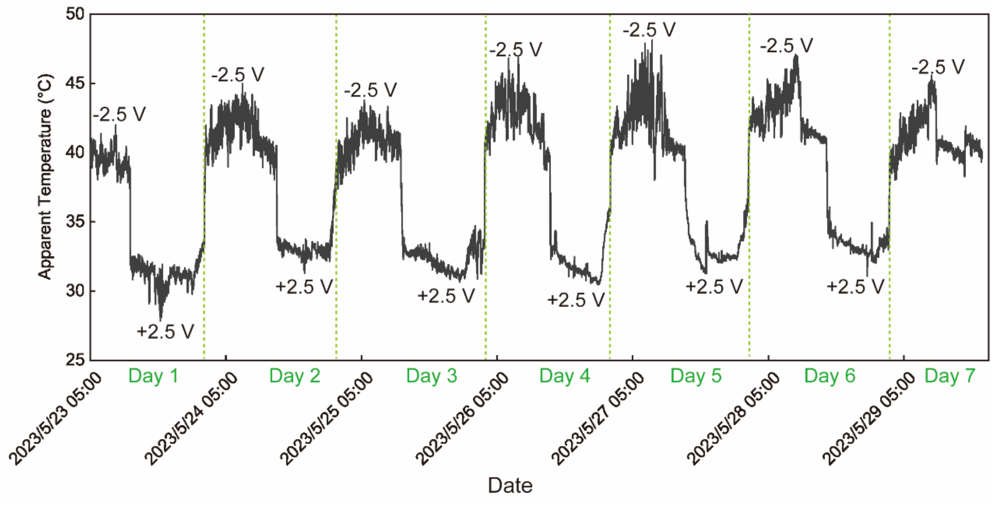

Supplementary Fig. 18 The long-term outdoor operation of TDIE regulator (Date: 2023.5.23 to 2023.5.29; Location: Changsha, China).

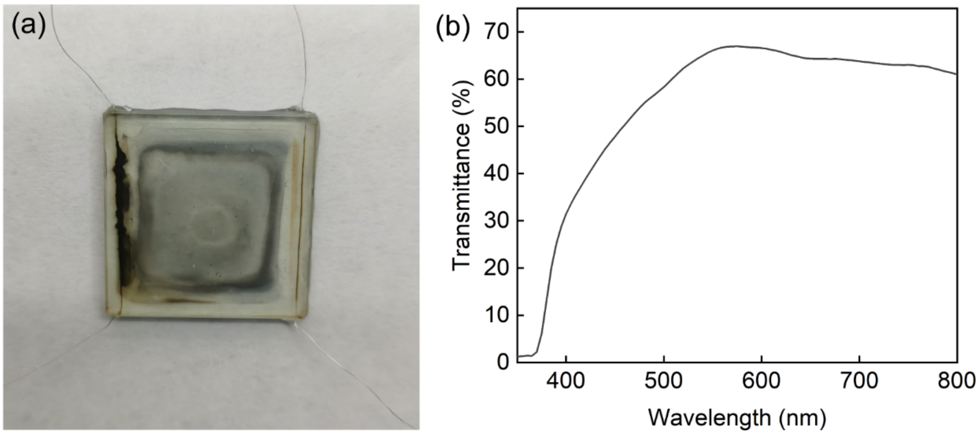

Supplementary Fig. 19 (a) the optical photo of TDIE regulator after long-term outdoor operation; (b) the transmittance of TDIE regulator after long-term outdoor operation.

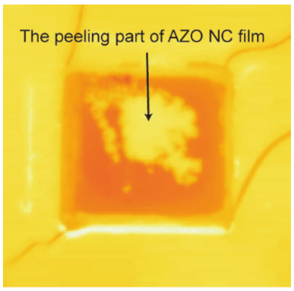

Supplementary Fig. 20 Infrared image of AZO NC film peeling after long-term operation of TDIE regulator.

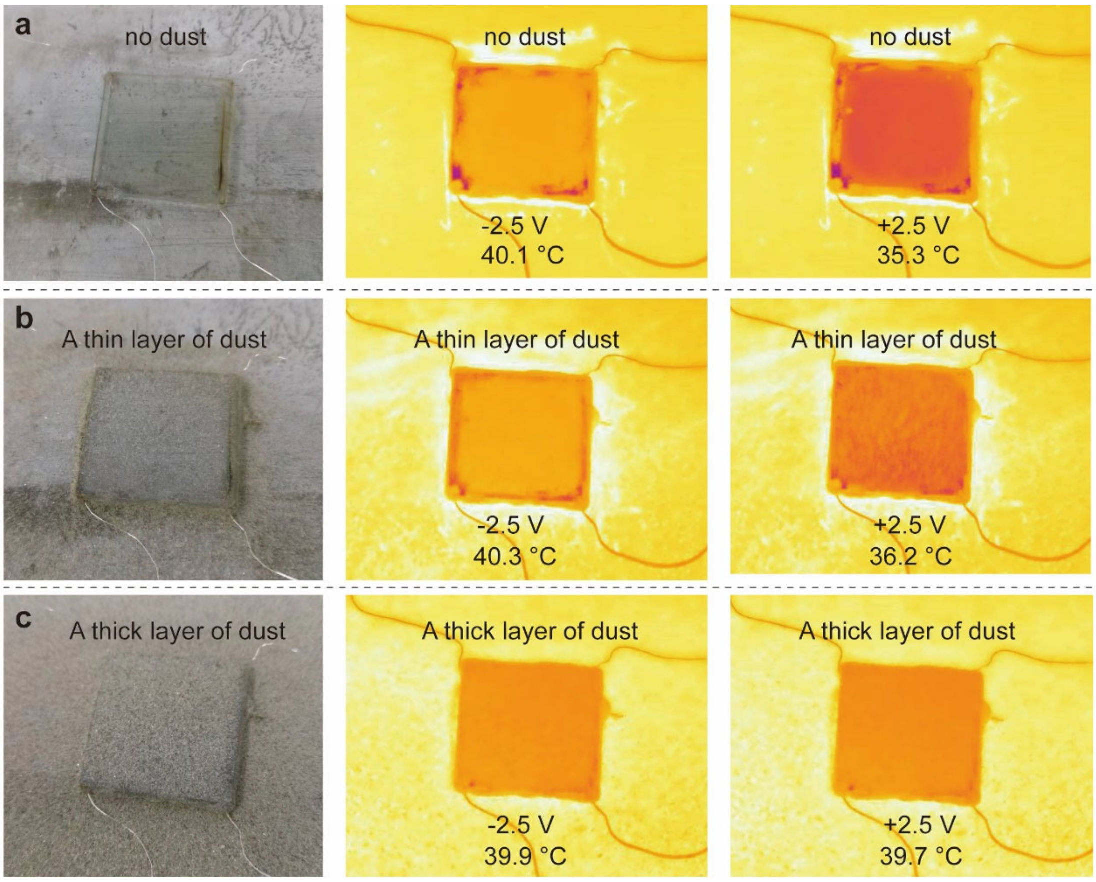

Supplementary Fig. 21 a, optical and infrared photos of the TDIE regulator without dust; **b**, optical and infrared photos of the TDIE regulator with a thin layer of dust on its surface; **c**, optical and infrared photos of the TDIE regulator with a thick layer of dust on its surface.

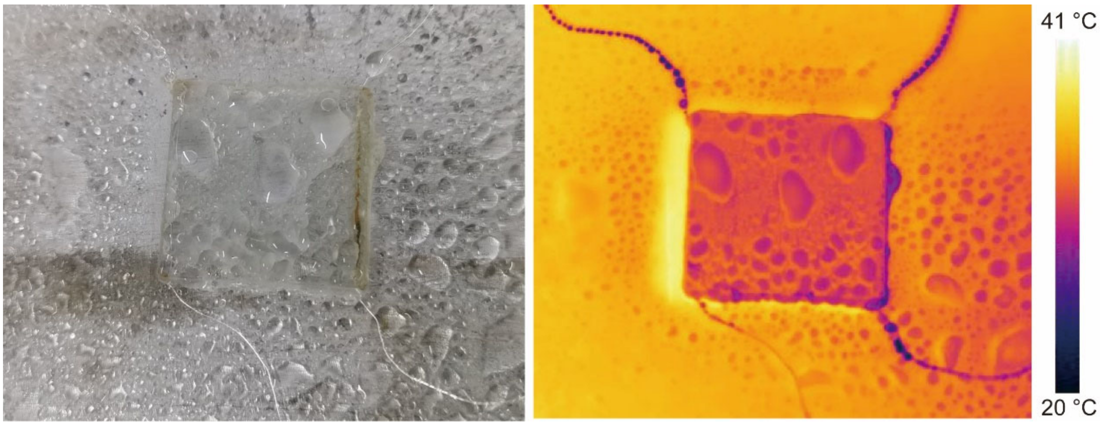

Supplementary Fig. 22 Optical and infrared photos of rainwater falling on the TDIE regulator.

Supplementary Note 5. Mechanism of infrared emissivity variation of AZO NCs.

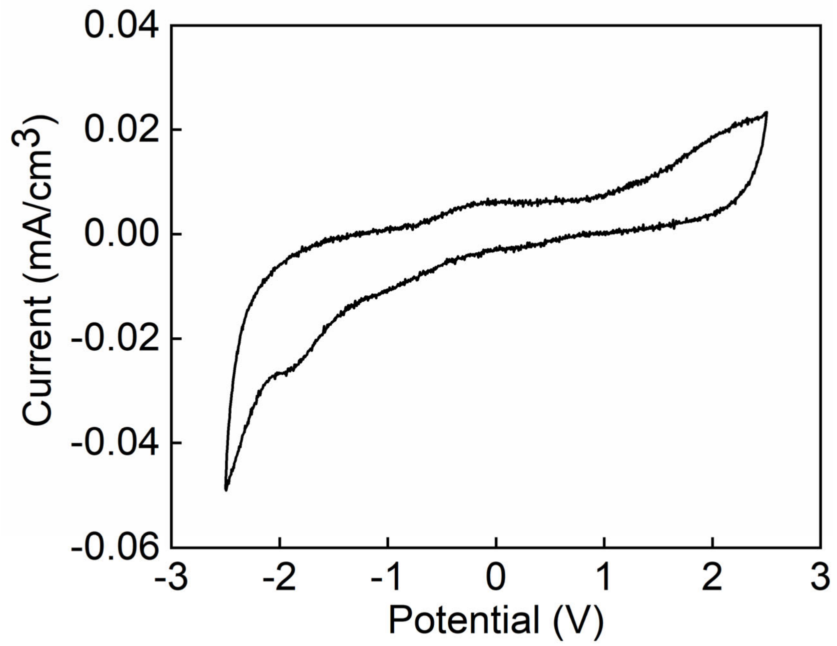

Supplementary Fig. 23 CV curve of AZO films in 1 M LiTFSI/tetraglyme electrolyte at 10 mV/s. The counter electrode and reference electrode are ITO glass.

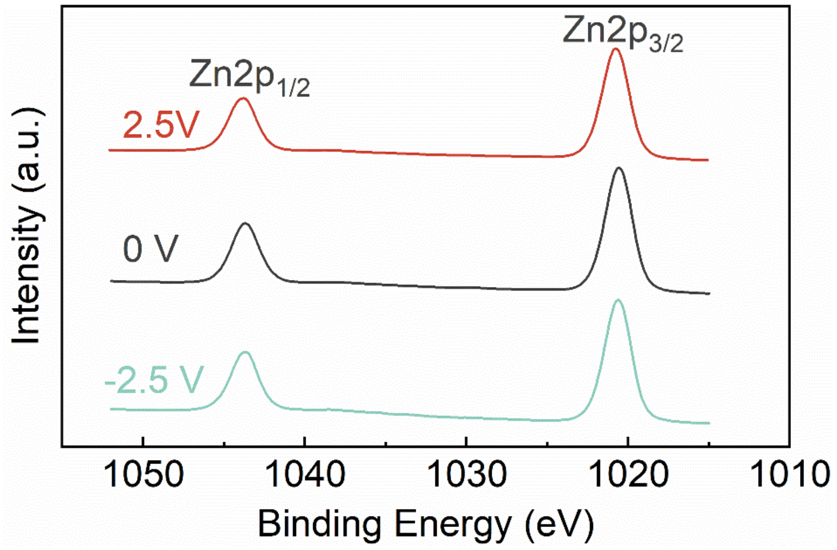

Supplementary Fig. 24 Analysis of Zn chemical state in AZO films using quasi-in-situ electrochemical XPS.

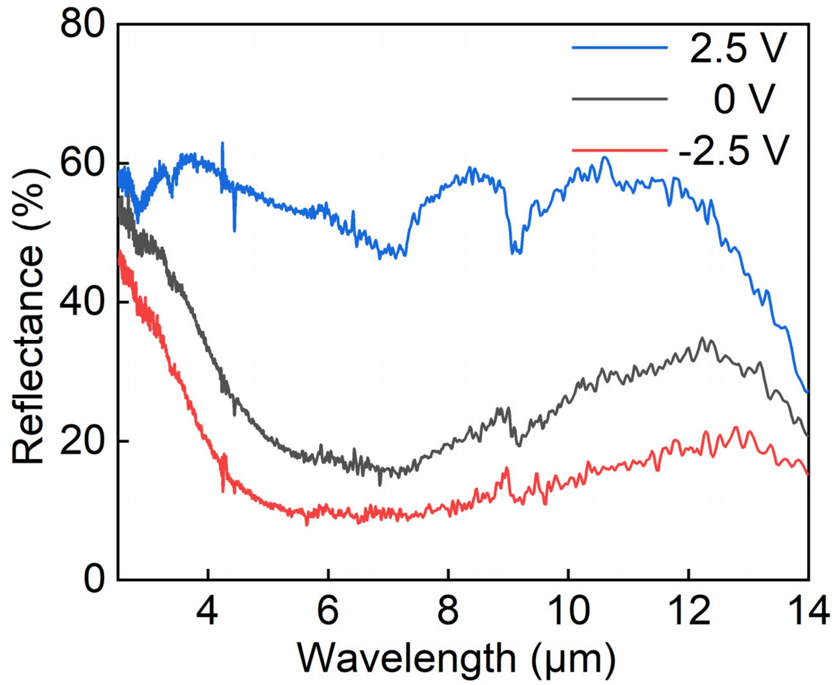

Supplementary Fig. 25 Infrared reflectance spectra of AZO film in 1M tetrabutylammonium perchlorate (TBAP) in acetonitrile electrolyte.

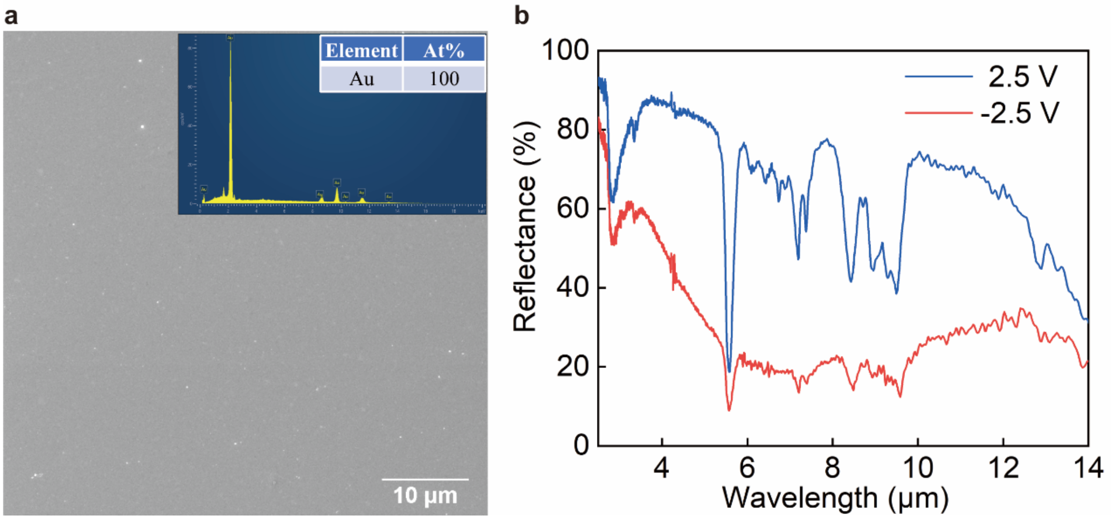

Supplementary Fig. 26 a, SEM image of dense gold film evaporated on AZO nanocrystals. **b,** Infrared reflectance spectra of devices prepared with gold film as the working electrode.

COMSOL Modeling Simulation

Schematic diagram of electron injection/ extraction in the surface depletion layer

of AZO nanocrystals was shown in Supplementary Fig. 27. The modelling and simulation process was based on the previously published studies[12-14]. Similarly, poisson's equation derived by Seiwatz[15] was solved numerically for spherical nanocrystals using a finite element method.

Supplementary Fig. 28 shows the band energetics of the AZO NCs used to solve the Poisson's equation (Equation S9)[16,17]. E_{ref} is the reference potential and the center of the band gap; E_{surf} is the applied surface potential relative to E_{ref}; E_{CB} (1.7 eV) is the bottom of the conduction band; E_{VB} (-1.7 eV) is top of the valence band; E_F (1.85 eV) is the Fermi level; and W_{sd} is the width of the surface depletion layer. When the surface potential is equal to the Fermi level (1.85 eV), the energy band does not bend. The potential below the Fermi level (1.85 eV) is the oxidation potential. The minimum oxidation potential was set as 0.8 eV, referring to previous literature[14]. The potential higher than the Fermi level is the reduction potential, and we set the surface potential of 2 eV as the reduction potential. Higher reduction potentials were not set, since the results did not converge when solving Poisson's equation for higher reduction potentials. Similar surface potential settings have also been reported in ITO nanoparticles[12, 14].

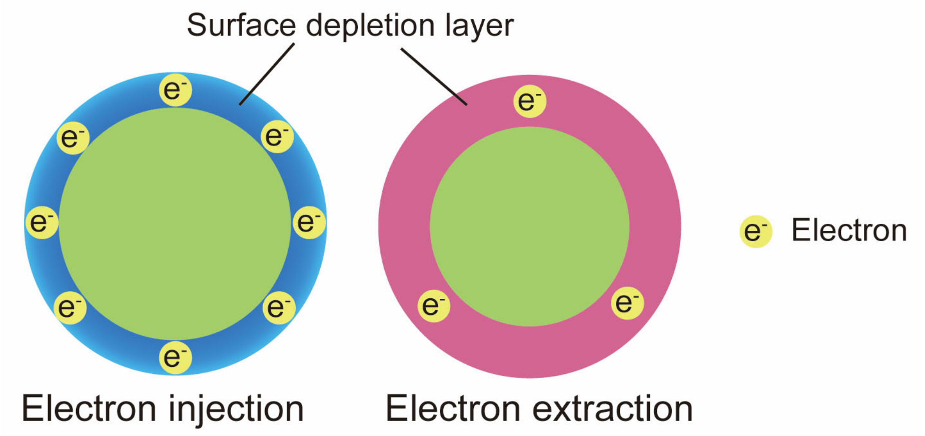

Supplementary Fig. 27 Schematic diagram of electron injection/ extraction in the surface depletion layer of AZO nanocrystals.

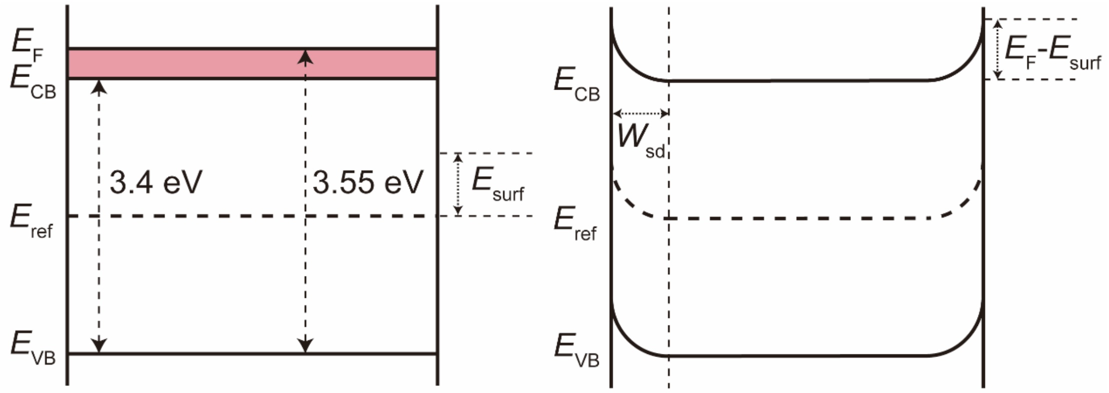

Supplementary Fig. 28 a, Band structure of AZO NCs. **b,** Band bending profiles due to the presence of surface depletion layers. The band bending is defined as E_F-E_{surf}.

Poisson's equation is shown in Equation (S9)

$$\nabla^2 u = -\frac{e^2 \rho}{\varepsilon \varepsilon_0 kT} \quad \text{(S9)}$$

The non-dimensional potential is defined as, $u = \frac{E_F - E_I}{kT}$, and k is the Boltzmann constant, ρ is the charge density, ε is the static dielectric constant, and ε_0 is the vacuum permittivity.

$$\rho = \rho_D - \rho_A + p - n \quad \text{(S10)}$$

where, ρ_D is the donor dopant density, ρ_A is the acceptor dopant density, p is hole density, and n is electron density. Because AZO NCs are n-type semiconductors, ρ_A=0.

The free electron concentration in the parabolic conduction band is calculated as

$$n = 4\pi \left[\frac{2m_e kT}{h^2}\right]^{\frac{3}{2}} \left[F_{\frac{1}{2}}(u - w_{C,I})\right] \quad \text{(S11)}$$

where $F_{\frac{1}{2}}(\eta) = \int_0^\infty \frac{x^{\frac{1}{2}} dx}{1+exp(x-\eta)}$, $w_{C,I} = \frac{E_{CB} - E_I}{KT}$

Similarly, hole concentration in the parabolic valence band is equal to

$$p = 4\pi \left[\frac{2m_h kT}{h^2}\right]^{\frac{3}{2}} \left[F_{\frac{1}{2}}(w_{V,I} - u)\right] \tag{S12}$$

where $w_{V,I} = \frac{E_{VB} - E_I}{KT}$

If the donor energy level is E_D, the activated dopant concentration can be expressed as,

$$\rho_D = \frac{N_D}{1 + 2exp(u - w_{D,I})} \tag{S13}$$

where $w_{D,I} = \frac{E_D - E_I}{KT}$

Substituting all the individual terms into Equation (S9)

$$\nabla^2 u = -\frac{e^2}{\varepsilon\varepsilon_0 kT}\left\{\frac{N_D}{1+2exp(u-w_{D,I})} + 4\pi\left[\frac{2m_h kT}{h^2}\right]^{\frac{3}{2}}\left[F_{\frac{1}{2}}(w_{V,I}-u)\right] - 4\pi\left[\frac{2m_e kT}{h^2}\right]^{\frac{3}{2}}\left[F_{\frac{1}{2}}(u-w_{C,I})\right]\right\} \tag{S14}$$

With the boundary condition,

$$u = u_{surf} = \frac{E_{surf} - E_I}{kT} \tag{S15}$$

Poisson's equation (Equation (S14)) was solved in COMSOL using a finite element scheme. The solution of Poisson's equation was used to calculate the band bending conditions and carrier concentration (Supplementary Fig. 29).

The carrier distribution in the NCs was not uniform owing to the presence of surface depletion layers. According to the Drude model, the dielectric constant of the nanocrystal surface differs from that of the interior. The effective dielectric function of the AZO NCs was calculated by discretizing the model sphere into core and multi shell structures (Supplementary Fig. 30), and then by multiple iterations of the core-shell Maxwell–Garnet effective medium theory reported by Zandi[14]. Using the effective dielectric function, the absorption of a single AZO NC was calculated using Mie theory

(Supplementary Fig. 31).

Subsequently, the effective dielectric function of the AZO film was calculated using the Maxwell–Garnett equation (Equation (S16))[18]. The AZO film was modelled using the COMSOL Electromagnetics Module and the extinction spectra of the AZO film at different surface potentials were fitted.

$$\frac{\varepsilon_{eff}-\varepsilon_m}{\varepsilon_{eff}+\varepsilon_m} = \varphi\frac{\varepsilon_r+\varepsilon_m}{\varepsilon_r+\varepsilon_m} \tag{S16}$$

where ε_{eff} is the effective dielectric function, φ is the volume fraction of the AZO NCs, ε_{m} is the environmental dielectric constant, ε_{r} is an effective dielectric function of the AZO NCs.

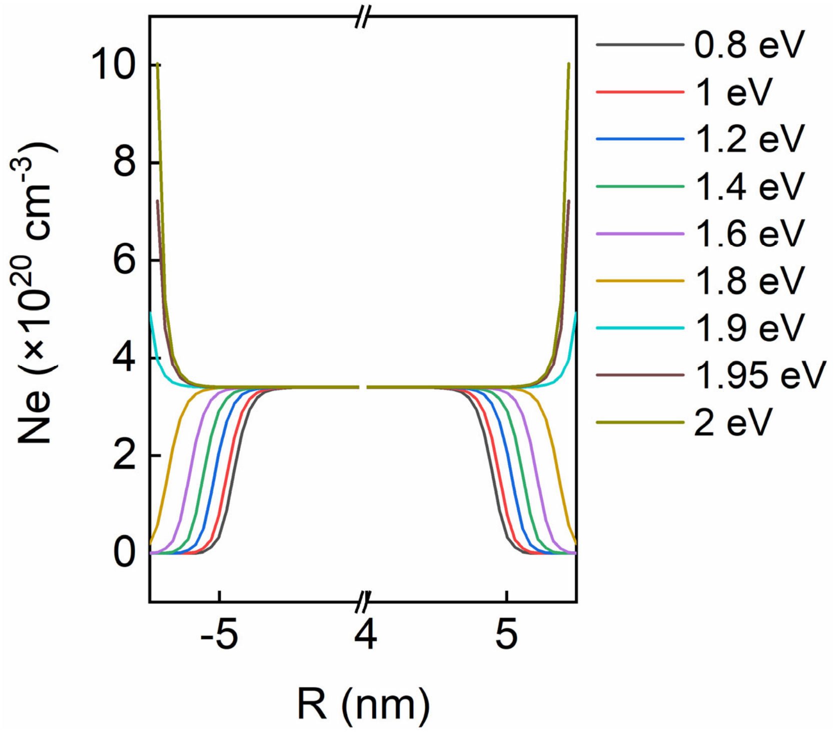

Supplementary Fig. 29 Radial carrier concentration of AZO NCs calculated by Poisson’s equation.

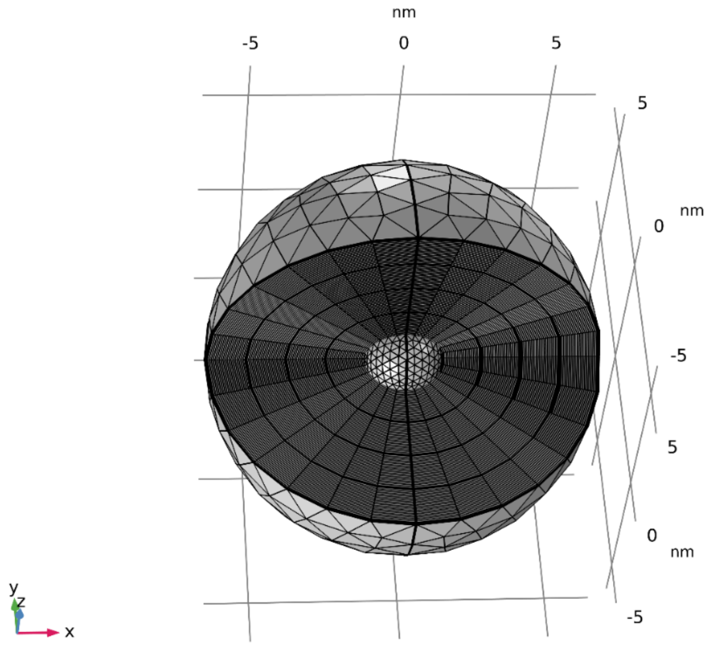

Supplementary Fig. 30 AZO NC model with a core and multi-shell configuration.

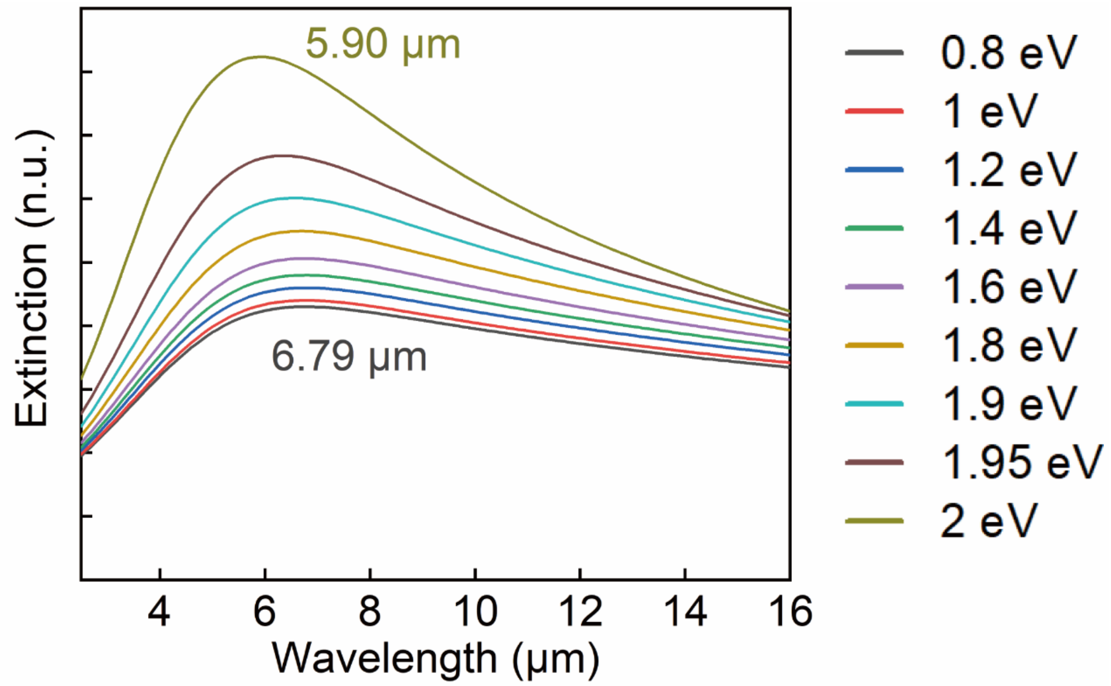

Supplementary Fig. 31 Extinction spectra of single AZO NCs calculated by Mie theory corresponding to different E_{surf}.

Supplementary Note 6. Outdoor radiation regulation of TDIE regulator.

We conducted an outdoor experiment to evaluate the performance of TDIE regulator. We measured the temperature change depend on the emissivity of TDIE regulator. The schematic diagram of the experimental setup is shown in supplementary Fig. 32. A chamber is dug out of the polystyrene foam box, and then the foam box is wrapped with Al foil. The TDIE regulator is covered above the chamber. The chamber was sealed using a low-density polyethylene (PE) film as a convection shield to reduce the effect of thermal convection on the sample. The optical photo of experimental setup is shown in supplementary Fig. 33. Since solar radiation has a significant effect on chamber temperature, we chose to conduct experiments at nighttime for investigating the impact of infrared radiation regulation of TDIE devices on chamber temperature. A control sample with a fixed emissivity (0.4 in 8-14 μm) is covered above the same chamber for comparison. In order to eliminate the influence of outdoor environmental temperature fluctuations over time, we calculated the temperature difference (ΔT) between the chamber temperature covered with TDIE regulator and the environment temperature. The change in ΔT is attributed to the regulation of infrared emissivity by the TDIE regulator. The average temperature change of the chamber is 0.61 °C, achieved by regulating infrared emissivity of TDIE regulator (supplementary Fig. 34). The ΔT between the chamber temperature covered with the fixed emissivity (0.4 in 8-14 μm) sample and the environment temperature is almost constant, which proves that the change of ΔT is due to the infrared emissivity regulation (supplementary Fig. 35).

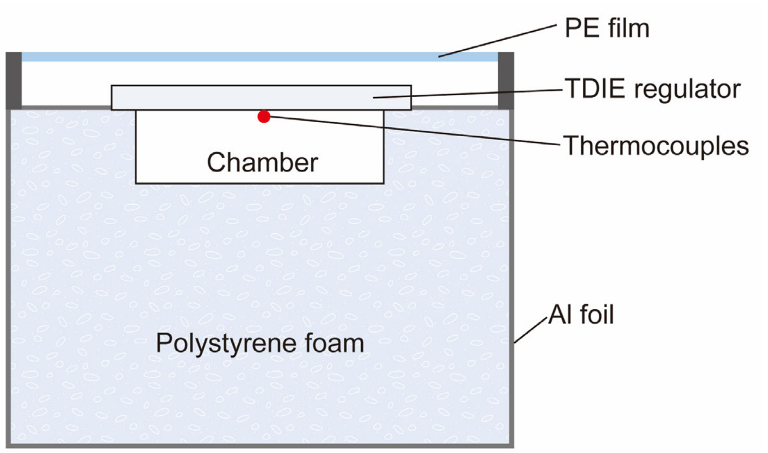

Supplementary Fig. 32 Schematic of the experimental setup. TDIE regulator was applied as a window of an enclosure chamber and the indoor temperature of the chamber was measured.

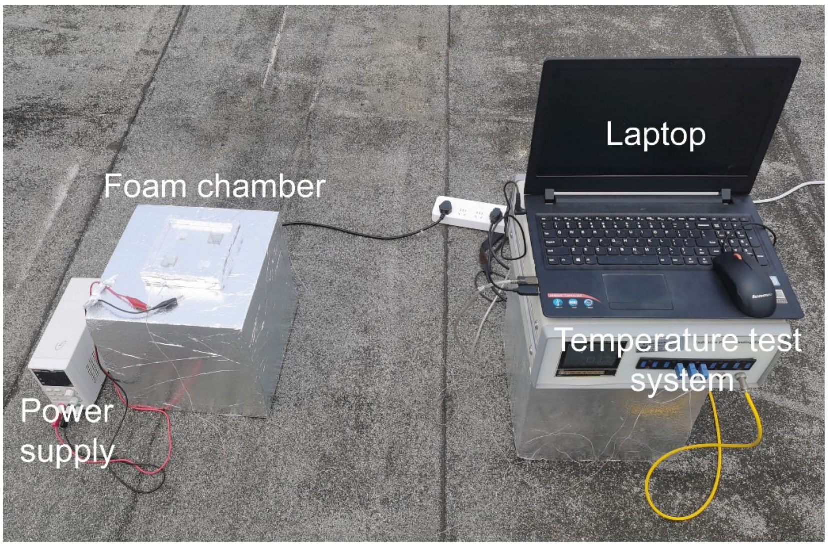

Supplementary Fig. 33 The optical photos of the experimental setup.

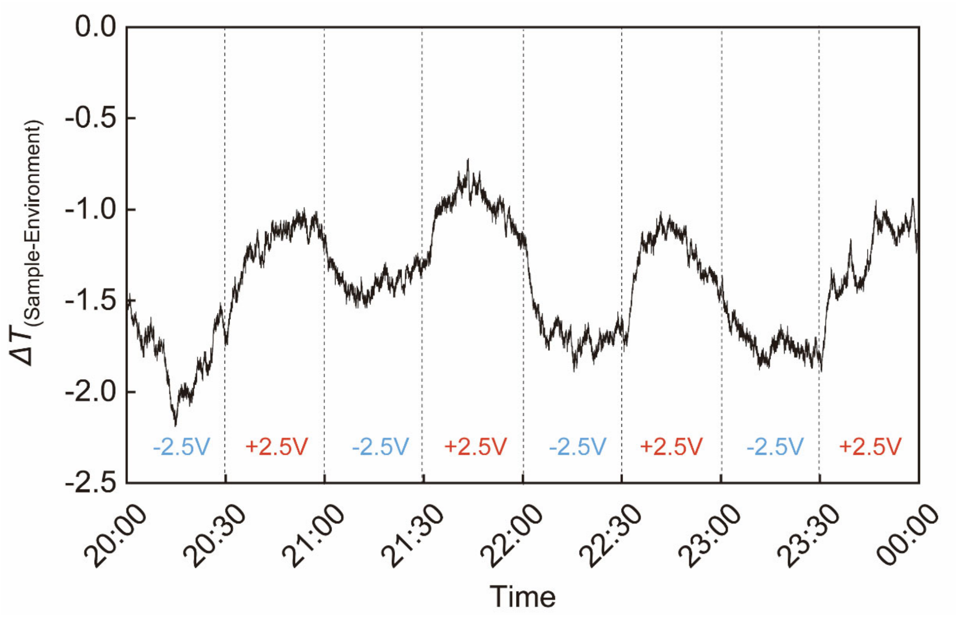

Supplementary Fig. 34 The temperature difference (*ΔT*) between the chamber temperature covered with TDIE regulator and the environment temperature. The voltage (±2.5 V) of the TDIE regulator is switched every half hour.

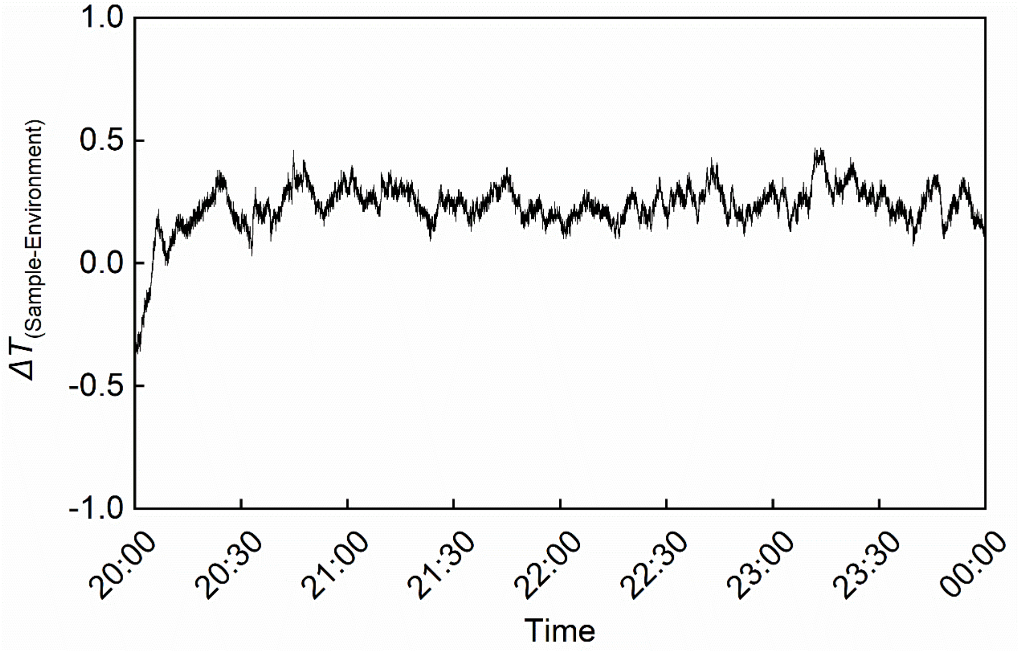

Supplementary Fig. 35 The temperature difference (*ΔT*) between the chamber temperature covered with control sample (fixed emissivity: 0.4) and the environment temperature.

We conducted an outdoor experiment to evaluate the infrared radiated power modulation performance of TDIE regulator. We measure the infrared radiated power modulation amount of TDIE regulator by power compensation method. The schematic diagram of the experimental setup is shown in Supplementary Fig. 36. The heating sheet is adhered to the back of the TDIE regulator. The thermocouple is sandwiched between the TDIE regulator and the heating sheet for temperature measurement. The chamber was sealed using a low-density polyethylene (PE) film as a convection shield to reduce the effect of thermal convection on the sample.

The optical photo of experimental setup is shown in Supplementary Fig. 37. The experimental setup was built on the roof. Since solar radiation has a significant effect on chamber temperature, we chose to conduct experiments at nighttime for investigating the infrared radiated power modulation of TDIE regulator. Two identical TDIE regulators, combined with heating sheet and thermocouples, were placed in the foam chamber. Before applying voltage to the TDIE regulator (21.15 to 21:25), the temperatures of the two TDIE regulators are basically equal (Supplementary Fig. 38). Two TDIE regulators are applied with voltages of +2.5V and -2.5V respectively (after 21:25), hereinafter named as TDIE(+) and TDIE(-), respectively. TDIE(+) and TDIE(-) showed a temperature difference of 0.62 °C caused by different emissivity of TDIE(+) and TDIE(-) (Supplementary Fig. 38). We applied a stepwise increase in heat input to the TDIE(-) using a heating sheet. The temperature of TDIE(-) increases gradually with the increase of heat input. When the heat input increased to 23.1 W/m^2, the temperature

of TDIE(-) was equal to that of TDIE(+), indicating that the experimental radiated power modulation amount of the TDIE regulator is ~23.1 W/m^2 (Supplementary Fig. 38).

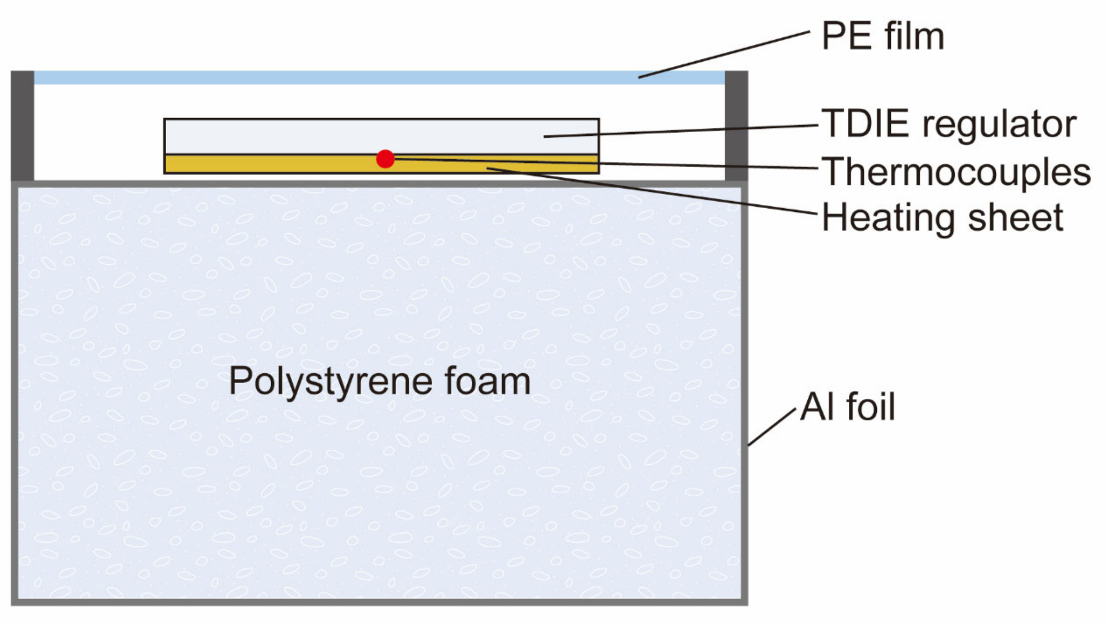

Supplementary Fig. 36 Schematic of the experimental setup.

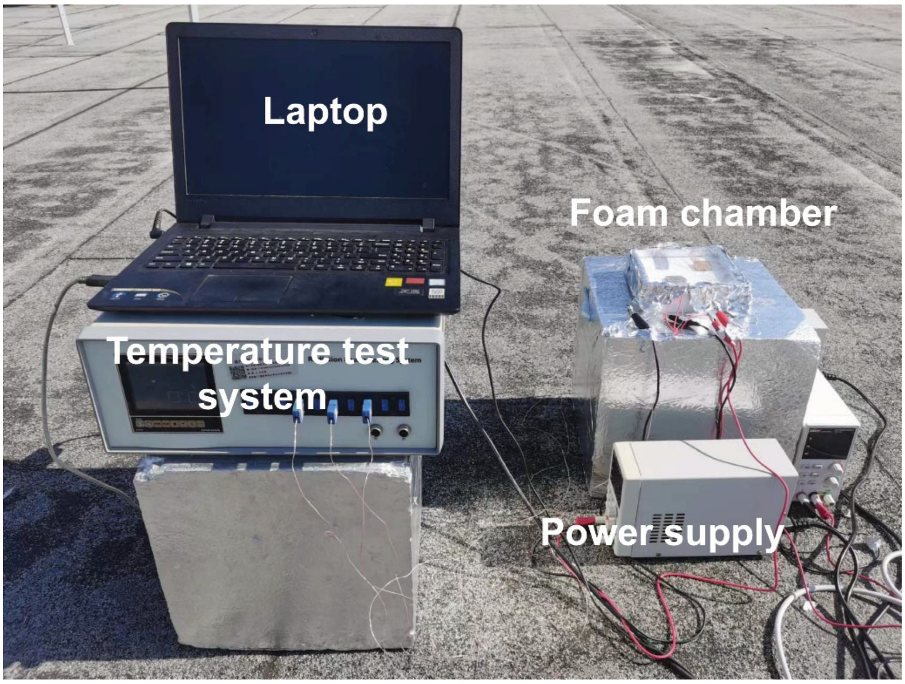

Supplementary Fig. 37 The optical photos of the experimental setup in roof. The power supply is used to provide the voltage for the TDIE regulator and provide energy

for the heating sheet for heat input.

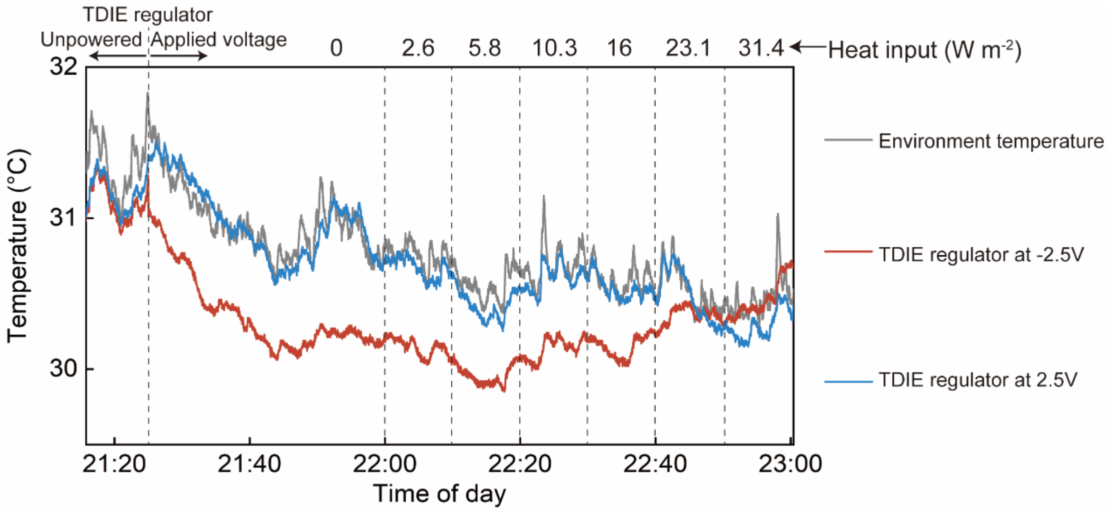

Supplementary Fig. 38 Temperature measurement of two TDIE regulators under applied voltage of 2.5 V and -2.5 V, respectively. The TDIE(-) input a stepwise increasing of heat from 22:00 (as indicated by the quantity displayed on the top of the graph).

Supplementary Note 7. Mode of SES roofs and windows and building HVAC energy saving performance simulation.

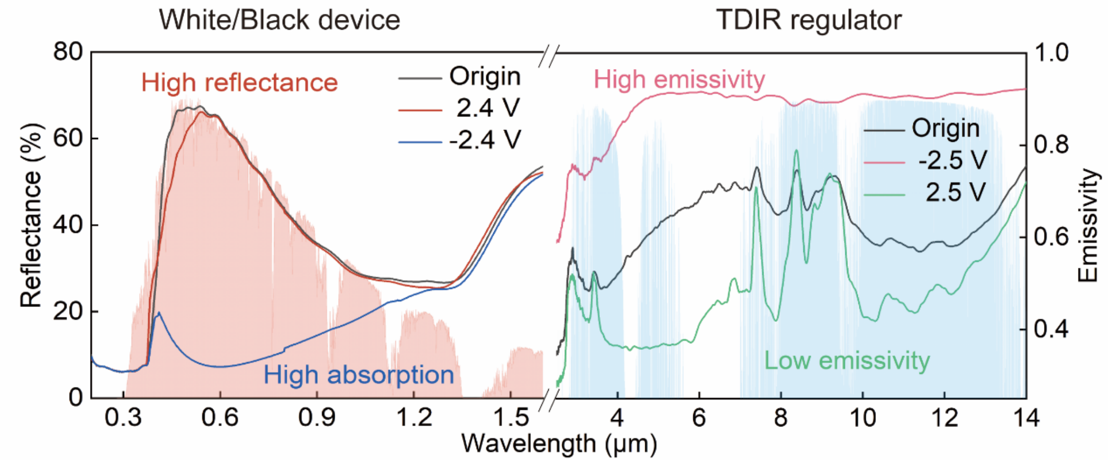

Supplementary Fig. 39 Visible and infrared independent regulation spectra of SES roofs. The preparation of White/Black device was introduced in Methods. The visible transmittance (400-760 nm) of White/Black device is 0, therefore the visible absorbance of White/Black device is 100%-reflectance. Due to the independent regulation capability of the White/Black device and TDIE regulator, the spectral curves of White/Black device and TDIE regulator at different voltages can be combined to achieve various operating modes. For example, Cooling mode 2 consists of the high reflectance curve of the White/Black device and the high emissivity curve of the TDIE regulator. The orange shaded area is the solar spectrum. The blue shaded area is the infrared atmospheric windows.

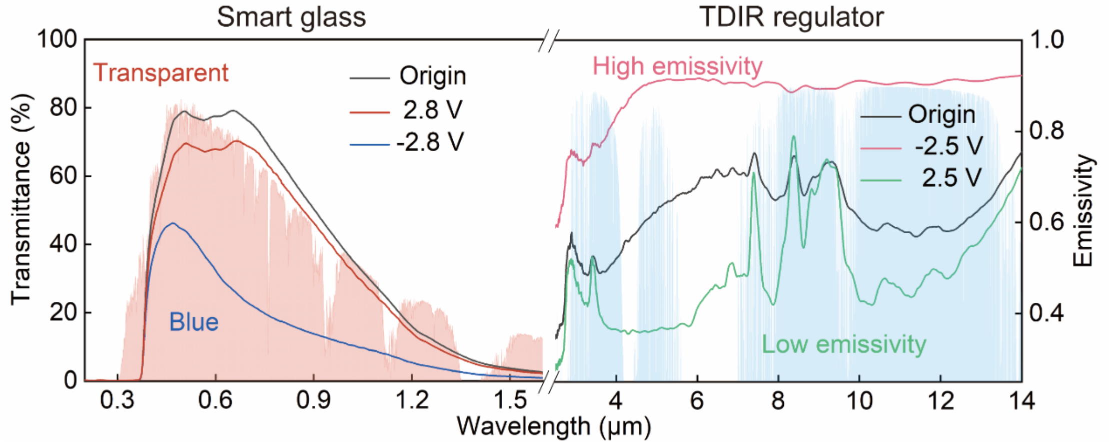

Supplementary Fig. 40 Visible and infrared independent regulation spectra of SES windows. The preparation of Smart glass was introduced in Methods. Due to the independent regulation capability of smart glass and the TDIE regulator, their spectra at different voltages can be combined to achieve a range of operating modes. For example, Cooling mode 2 consists of the blue curve of smart glass and the high emissivity curve of TDIE regulator. The orange shaded area is the solar spectrum. The blue shaded area is the infrared atmospheric windows.

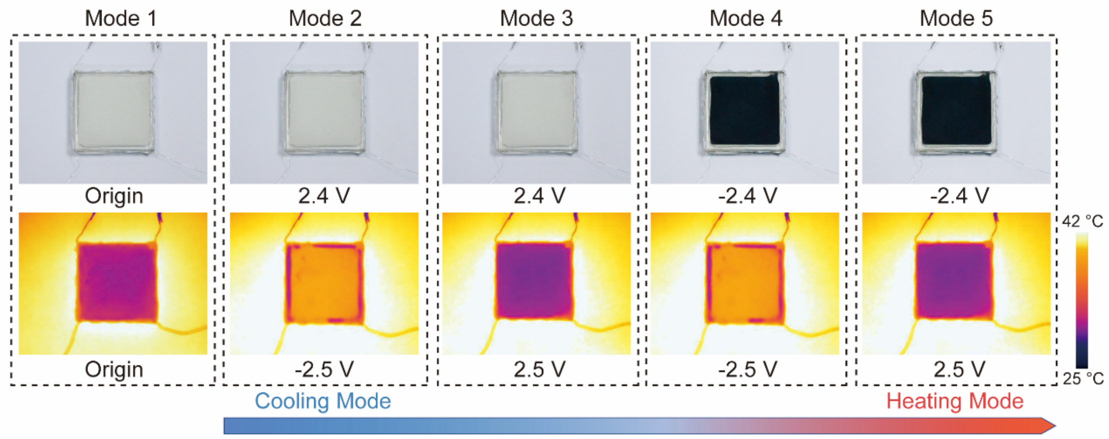

Supplementary Fig. 41 Different modes of SES roofs. TDIE regulator and White/Black device can be regulated independently. Mode 2 is the cooling mode (high visible reflectivity, high infrared emissivity), and Mode 5 is the heating mode (high

visible absorbance, low infrared emissivity).

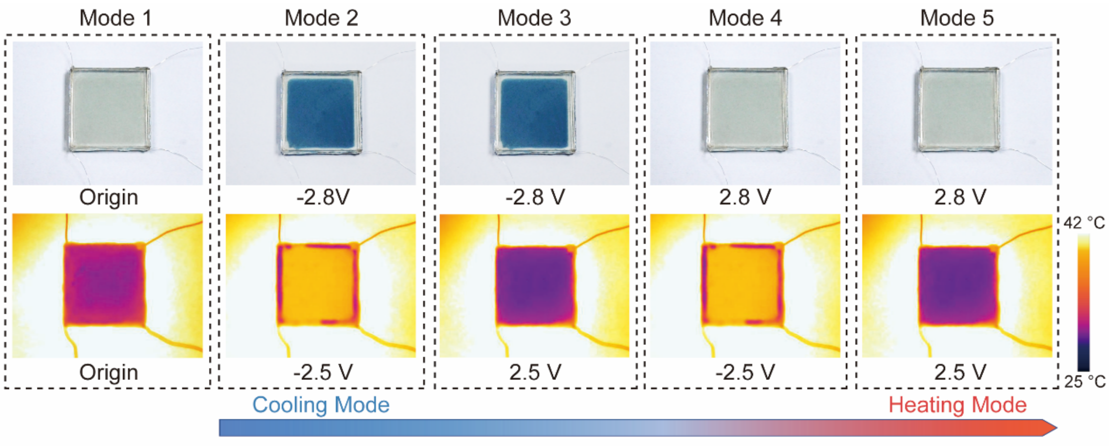

Supplementary Fig. 42 Different modes of SES windows. TDIE regulator and smart glass can be regulated independently. Mode 2 is the cooling mode (low visible transmittance, high infrared emissivity), and Mode 5 is the heating mode (high visible transmittance, low infrared emissivity).

Building HVAC energy saving performance simulation

The HVAC energy consumption of SES buildings was calculated using *EnergyPlus* software. The optical and infrared emissivity properties of SES windows and roofs were imported into *EnergyPlus* as parameters for the windows and roofs of the building model. A simple program was designed in *EnergyPlus* for the cooling and heating modes of the SES buildings. If the ambient temperature was lower than 21 °C, the heating mode (mode 2; Supplementary Fig. 41, Supplementary Fig. 42) of the SES windows and roof was applied. If the ambient temperature was lower than 21 °C, the heating mode (mode 5; Supplementary Fig. 41, Supplementary Fig. 42) of the SES windows and roof was used. The HVAC energy-saving performance of the SES building was calculated by comparing the performance of the building model with

normal window glass and roof under the same operating conditions The optical and infrared data used in the simulations are presented in Supplementary Table 2.

The Medium Office prototype building model was used in the simulation, which is derived from Commercial Reference Building Models of the U.S. Department of Energy[19], in compliance with ASHRAE Standard 90.1 Prototype Building Models[20]. The building area is 4982.19 m^2 with a 33% window to wall ratio. The Medium Office building specifications of the prototype are displayed in Supplementary Table 3.

The 30 climate zones of the world (Köppen-Geiger climate classification[21]) are divided into 10 categories according to the average annual temperature. Ten cities (Singapore, Honolulu, Turpan, Tokyo, London, Reykjavik, Beijing, Oslo, Anchorage, Yakutsk) are selected to represent 30 climate zone and the specific classification method is shown in Supplementary Table 4. Furthermore, additional cities across different climate zones around the world were adopted for calculating average annual individual building HVAC energy savings: Miami, USA (Am); Rio de Janeiro, Brazil (Aw); Cairo, Egypt (Bwh); Zaragoza, Spain (BSk); Rome, Italy (Csa); Hong Kong, China (Cwa); Cape Town, South Africa (Csb); Kunming, China (Cwb); Berlin, Germany (Cfb); Toronto, Canada (Dfa); Salt Lake City, USA (Dsa); Calgary, Canada (Dwb); Moscow, Russia (Dfb); Fairbanks, USA (Dfc); Mohe, China (Dwc). The average annual individual building HVAC energy savings and the savings percentage (compared to standard buildings) in different cities were calculated (Supplementary Fig. 43).

The reduction in carbon emissions from the use of SES buildings can be calculated

(in terms of coal g CO_2 equivalent/kWh) by the following equation[22]:

$$E_{sav} = 820 \times \Delta E_b \tag{S17}$$

where ΔE_b is the year-round individual building HVAC energy saving of SES buildings compared to normal buildings using the Medium Office prototype building model (1MBtu=293.2 kWh).

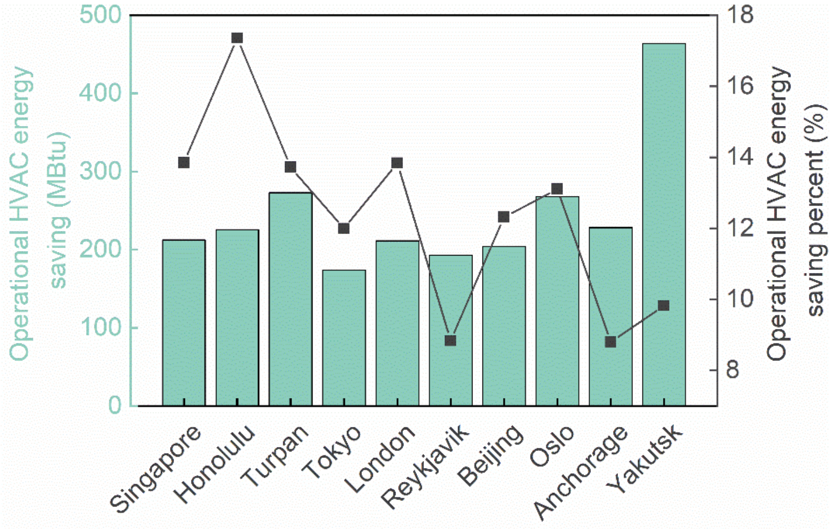

Supplementary Fig. 43 Annual operational HVAC energy savings and savings rate of SES buildings in various cities.

Supplementary Note 8. TDIE regulator applications in spacecraft thermal management.

Radiative heat transfer to deep space through infrared thermal radiators is the sole mode for spacecraft to dissipate heat[23]. Because there is no limitation to the infrared atmospheric window, TDIE regulators can achieve infrared radiation power modulation of 85.4 W/m^2 at 298K. Compared to mechanical louvers[24], TDIE regulators have simpler structures and lower drive voltages (<2.5 V).

In a vacuum environment, the surface temperature of an object exposed to space depends largely extent on the ratio between the solar absorbance (α_s) and infrared emissivity (ε) of the surface; and thus, the different surface temperatures of the spacecraft can be regulated by different α_s/ε ratios (Equation (S18), Equation (S19)). Owing to their transparency, TDIE regulators can be compatible with spacecraft thermal control films[25] to achieve different α_s/ε value and achieve the specific surface-temperature requirement of a spacecraft.

An adiabatic plane (such as the adiabatic surface on a spacecraft) was set, which was vertically exposed to the sun. If there is no other heat source, the thermal equilibrium temperature can be calculated by:

$$T = \sqrt[4]{\frac{\alpha_s S}{\varepsilon\sigma}} \tag{S18}$$

where S is the solar constant (1367±7 W/m^2), and σ is Stefan–Boltzmann constant (5.67×10^{-8} W/m^2/K^4).

If the spacecraft is assumed to be an isothermal sphere, the thermal equilibrium

temperature of the surface is:

$$T = \sqrt[4]{\frac{\alpha_s S}{\varepsilon 4\sigma}} \quad \text{(S19)}$$

Therefore, the surface temperature of either an adiabatic plane or an isothermal sphere depends on the α_s/ε ratio.

Supplementary Note 9. Circuit design and program of visible-transparent infrared displays.

Supplementary Fig. 44 shows the circuit diagram of visible-transparent infrared displays. The circuit contains an AT89C52 microcontroller and eighteen 74HC573 latches. The AT89C52 microcontroller is used to run the program and control the signal inputs of 74HC573 latches (Supplementary Fig. 45). The output of ±2.5V is controlled by a 74HC573 latch (Supplementary Fig. 46).

74HC573 latch work as below：

OE	Dx(x is 0~7)	Qx (x is 0~7)
0	0	/
	1	/
1	0	0
	1	1

Two states of the output port are shown below:

U2-Qx(x is 0~7)	U3-Qx(x is 0~7)	NMOS	PMOS	Volts
0	1	on	off	-2.5V
1	0	off	on	+2.5V

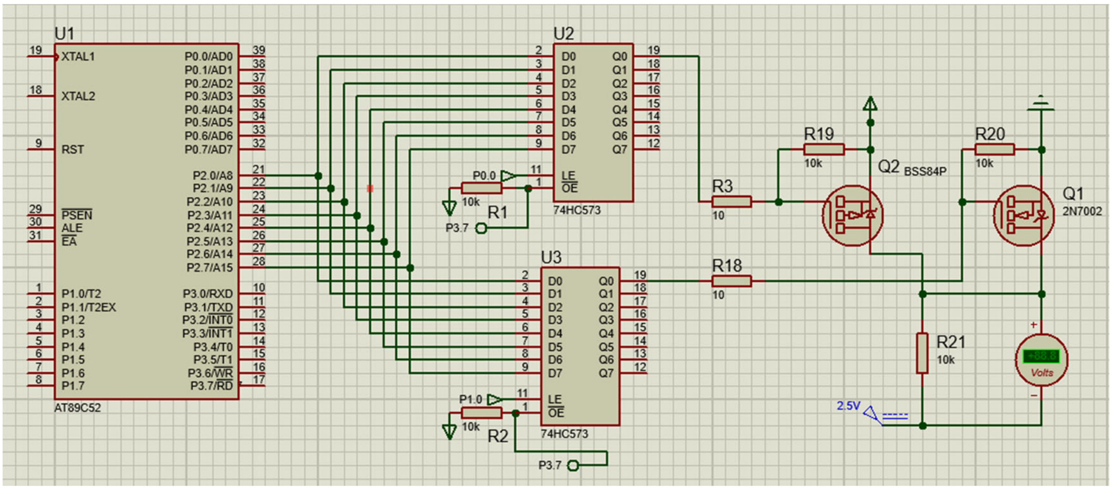

Supplementary Fig. 44 Control circuit of the infrared display. Note that the circuit diagram only shows the AT89C52 microcontroller controlling the signal input of two 74HC573 latches, i.e. one pixel in the infrared display.

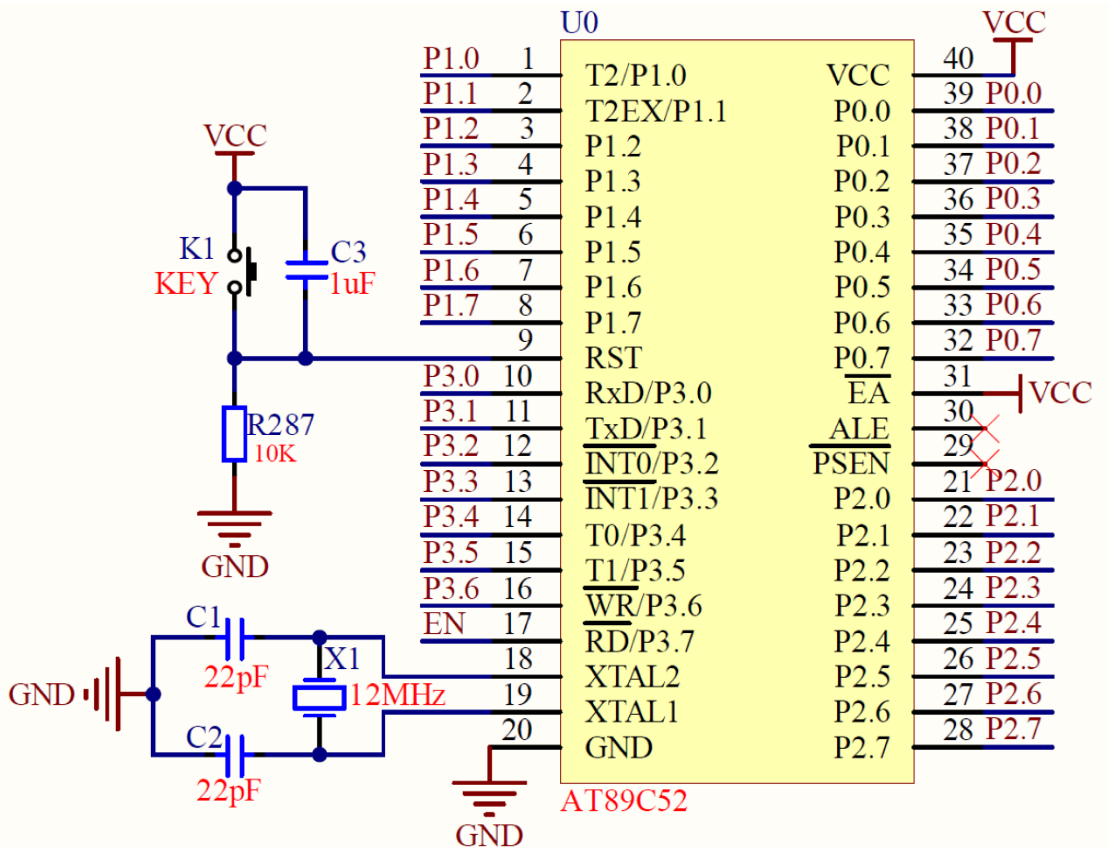

Supplementary Fig. 45 Running the program of AT89C52 microcontroller.

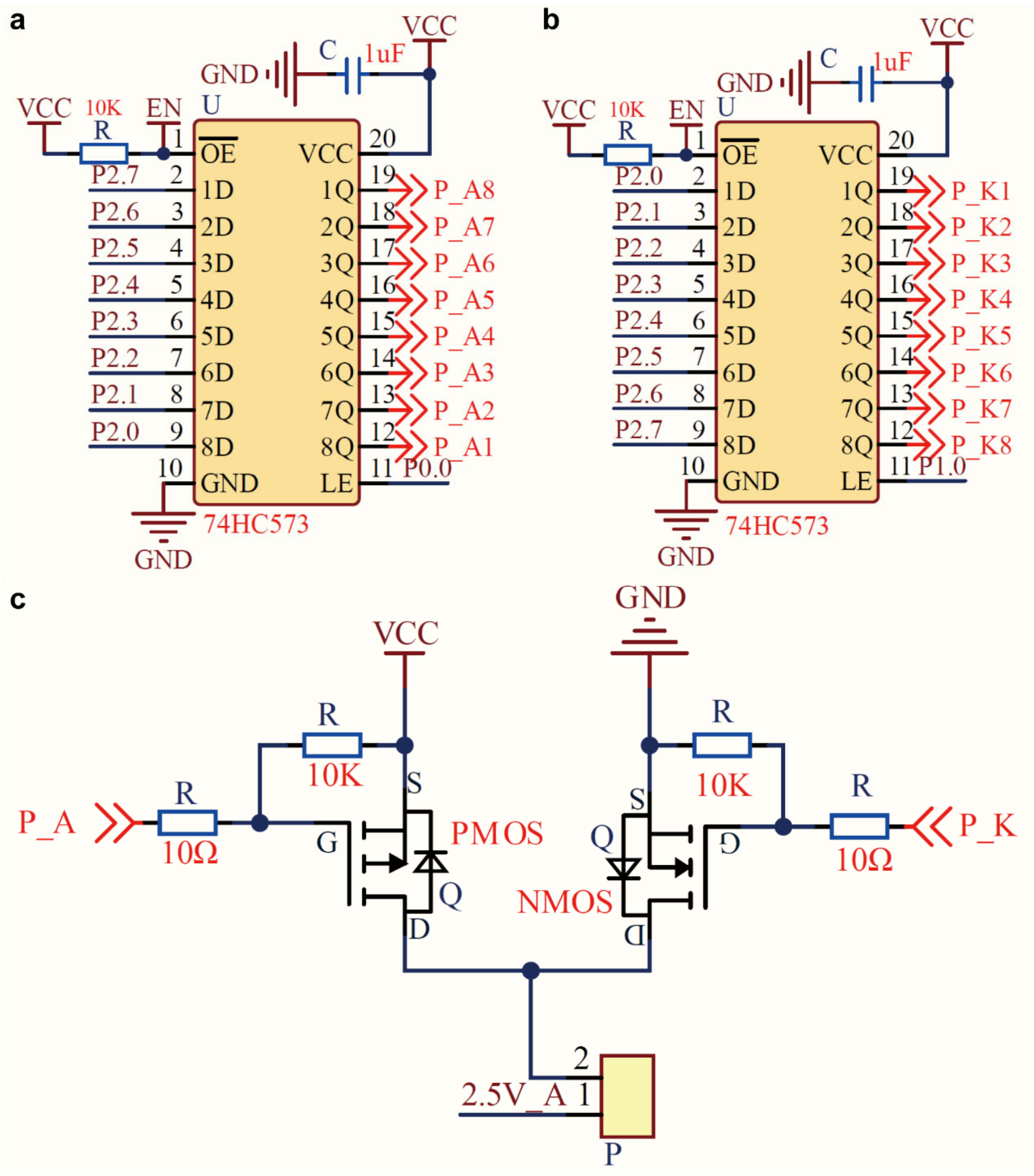

Supplementary Fig. 46 a, Addressing and controlling the output of pulse signal A using 74HC573 latch. **b,** Addressing and controlling the output of pulse signal K using 74HC573 latch. **c,** Output of ± 2.5V voltage is realized by pulse signals A and K (A: 2.5V, K: -2.5V).

Kiel uVision4 software was used for programming, and the program was downloaded to STC89C52RC microcontroller through stc-isp-15xx-v8.65. By

changing the numbers (1, 2) in the matrices u8 a1 [30], u8 b1 [30], u8 c1 [30], different animations of infrared display can be demonstrated. The animation code is shown below:

```
#include "reg51.h"
#include<intrins.h>
typedef unsigned int u16;
typedef unsigned char u8;
sbit en = P3^7;
sbit clk_p1 = P0^0;
sbit clk_n1 = P1^0;
sbit clk_p2 = P0^1;
sbit clk_n2 = P1^1;
sbit clk_p3 = P0^2;
sbit clk_n3 = P1^2;

u8 a1[30]={ 1,2,2, 2,1,2, 2,2,1, 2,1,2, 1,2,2, 2,1,2, 2,2,1, 2,1,2} ;
u8 b1[30]={ 1,2,2, 2,1,2, 2,2,1, 2,1,2, 1,2,2, 2,1,2, 2,2,1, 2,1,2};
u8 c1[30]={ 1,2,2, 2,1,2, 2,2,1, 2,1,2, 1,2,2, 2,1,2, 2,2,1, 2,1,2};
u8 Time = 2;
void HC573_int()
{
    en=1;
    P2=0xff;
    P0=0xff;
    _nop_();
    P0=0x00;

    P2=0x00;
    P1=0xff;
    _nop_();
    P1=0x00;
    en=0;
}
void delay(u16 nus)
{
    while(nus--);
}
void main()
{
```

```
u8 i;
HC573_int();
while(1)
{
    P2=0xFF;
    for(i=0;i<3;i++)
    {
        if(a1[i]==1)
        {
            P2&=(~(0x01<<i));
        }
    }
    clk_p1=1;
    delay(10);
    clk_p1=0;

    P2=0x00;
    for(i=0;i<3;i++)
    {
        if(a1[i]==2)
        {
            P2|=(0x01<<i);
        }
    }
    clk_n1=1;
    delay(10);
    clk_n1=0;
```

The different animations are implemented by means of the following instructions:

```
u8 a1[30]={2,2,2, 1,2,2, 2,1,2, 2,2,1, 2,2,2, 2,2,2, 2,2,2, 2,2,2, 2,2,2, 2,2,2};
u8 b1[30]={2,2,2, 2,2,2, 2,2,2, 2,2,2, 1,2,2, 2,1,2, 2,2,1, 2,2,2, 2,2,2, 2,2,2};
u8 c1[30]={2,2,2, 2,2,2, 2,2,2, 2,2,2, 2,2,2, 2,2,2, 2,2,2, 1,2,2, 2,1,2, 2,2,1};

u8 a1[30]={2,1,2, 1,2,1, 2,1,2, 1,2,1, 2,1,2, 1,2,1, 2,1,2, 1,2,1, 2,1,2, 1,2,1};
u8 b1[30]={1,1,1, 2,1,2, 1,1,1, 2,1,2, 1,1,1, 2,1,2, 1,1,1, 2,1,2, 1,1,1, 2,1,2};
u8 c1[30]={2,1,2, 1,2,1, 2,1,2, 1,2,1, 2,1,2, 1,2,1, 2,1,2, 1,2,1, 2,1,2, 1,2,1};

u8 a1[30]={2,1,2, 1,2,2, 2,2,2, 2,2,1, 2,1,2, 1,2,2, 2,2,2, 2,2,1, 2,1,2, 1,2,2};
u8 b1[30]={2,2,2, 2,2,2, 1,2,1, 2,2,2, 2,2,2, 2,2,2, 1,2,1, 2,2,2, 2,2,2, 2,2,2};
u8 c1[30]={2,1,2, 2,2,1, 2,2,2, 1,2,2, 2,1,2, 2,2,1, 2,2,2, 1,2,2, 2,1,2, 2,2,1};

u8 a1[30]={1,2,2, 2,1,2, 2,2,1, 2,2,2, 2,2,2, 2,2,2, 2,2,1, 2,1,2, 1,2,2, 2,2,2};
```

```
u8 b1[30]={2,2,2, 1,2,2, 2,1,2, 2,2,1, 2,2,2, 2,2,1, 2,1,2, 1,2,2, 2,2,2, 2,2,2};
u8 c1[30]={2,2,2, 2,2,2, 1,2,2, 2,1,2, 2,2,1, 2,1,2, 1,2,2, 2,2,2, 2,2,2, 2,2,2};

u8 a1[30]={ 1,2,2, 2,1,2, 2,2,1, 2,1,2, 1,2,2, 2,1,2, 2,2,1, 2,1,2, 1,2,2, 2,1,2};
u8 b1[30]={ 1,2,2, 2,1,2, 2,2,1, 2,1,2, 1,2,2, 2,1,2, 2,2,1, 2,1,2, 1,2,2, 2,1,2}
u8 c1[30]={ 1,2,2, 2,1,2, 2,2,1, 2,1,2, 1,2,2, 2,1,2, 2,2,1, 2,1,2, 1,2,2, 2,1,2};
```

Supplementary Note 10. Adaptive visible-infrared compatible camouflage.

The current electrically controlled DIE regulators are black or opaque in color[7,26-28], making them incompatible with traditional camouflage equipment and limiting their application in the field of camouflage. TDIE regulators can independently control infrared emissivity, and their high transparency ensures that they do not interfere with conventional optical camouflage equipment, effectively realizing visible-infrared compatible camouflage. Moreover, we can realize adaptive visible-infrared compatible camouflage by coating an electrochromic device with a TDIE regulator, which changes its visible and infrared characteristics depending on the background environment (Supplementary Fig. 47).

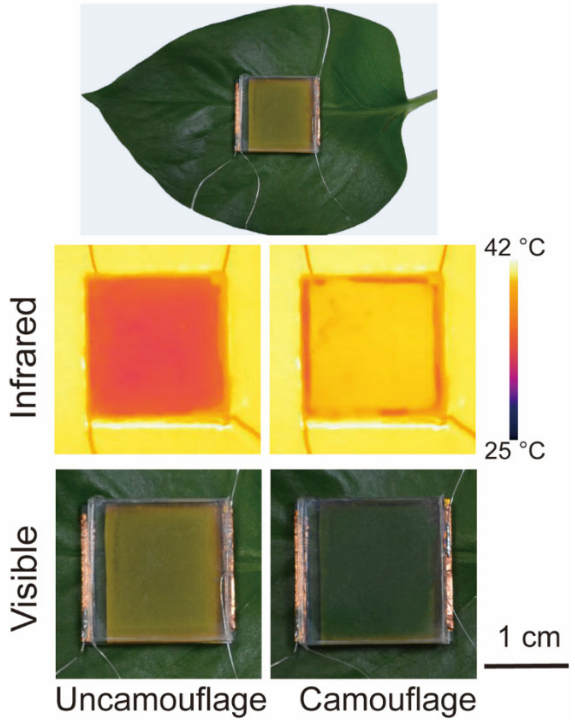

Supplementary Fig. 47 Demonstration of adaptive visible-infrared compatible camouflage.

Supplementary Table 1 Comparison of different infrared emissivity regulation devices.

Materials	Work mechanism	Infrared emissivity regulation		Response time	Cycling stability	Spectral range	Color	Ref.
		MWIR	LWIR					
Dielectric elastomer actuator	Electrical or mechanical	0.28	0.27	<1 s	>750	2.5–15 μm	Silver	[29]
Bioinspired composite material	mechanical	-	0.33	-	-	5–16 μm	Gold	[30]
Reconfigurable graphene device	Mechanical	0	0.265	-	>30	7.5–15 μm	-	[31]
GST	Thermochromic	-	0.324	Several seconds	-	7.5–13 μm	-	[32]
	Thermochromic	-	~0.7	~60 s	-	7.5–13 μm	Yellow	[10]
VO_2	Thermochromic	-	0.47	<2 s	-	2.5–45 μm	Silver	[33]
	Thermochromic	-	0.26	-	-	1.6-20 μm	62% visual transmittance	[34]
	Thermochromic	-	0.4	-	-	2.5–15 μm	Translucent	[35]
$W_xV_{1-x}O_2$	Thermochromic	-	0.7	-	-	6–16 μm	Gold	[36]
Solid-state electrochromic	Electrochromic	0.52	0.78	≥4 min	-	2–26 μm	Green	[5]
WO_3	Electrochromic	0.727	0.153	180 s	-	2.5–13 μm	Blue	[6]
$Li_4Ti_5O_{12}$	Electrochromic	0.68	0.315	1 min	100	Visible and infrared	White and black	[7]
PANI	Electrochromic	0.11	0.436	<2 s	500	2.5–18 μm	Yellow and black	[26]
PANI	Electrochromic	0.183	0.388	-	-	2.5–25 μm	Black	[37]
Graphene	Electrochromic	-	0.45	1 s	2200	Visible, infrared, and microwave	Gold and black	[27]

**Materials**	**Work mechanism**	**Infrared emissivity regulation**		**Response time**	**Cycling stability**	**Spectral range**	**Color**	**Ref.**
		**MWIR**	**LWIR**					
Multiwalled carbon nanotubes	Electrochromic	-	0.7	1 s	3500	7.5–13 μm	Black	[28]
Ag	Reversible electrodeposition	0.77	0.71	≤15 s	≥350	Visible, infrared	Black	[8]
Cu	Reversible electrodeposition	-	0.85	-	2500	Visible, infrared	-	[38]
Quantum wells	Electron injection and extraction		0.5	10 kHz	-	9.1–10 μm	-	[39]
Graphene resonator	Electron injection and extraction	0.02		2 kHz	-	6.25–8.3 μm	-	[40]
**AZO NCs**	**Electron injection and extraction**	**0.51**	**0.41**	**<600 ms**	**10,000**	**Infrared**	**Transparent**	**This work**

Supplementary Table 2 Optical and infrared properties of the samples used in the simulation. All spectral data of the SES windows were entered into *EnergyPlus* software and are therefore not shown here.

Parameters	SES Roofs		Normal roof	Normal glass
	Mode 2	Mode 5		
$\varepsilon_{\text{LWIR-Front}}$	0.91	0.51	0.9	0.84
$\varepsilon_{\text{LWIR-Back}}$	None	None	None	0.84
A_{lum}	0.43	0.84	0.7	None
T_{lum} (%)	None	None	None	88.1
$R_{\text{lum Front and back}}$ (%)	None	None	None	8
A_{sol}	0.53	0.79	0.7	None
T_{sol} (%)	None	None	None	77.5
$R_{\text{sol Front and back}}$ (%)	None	None	None	7.1

Supplementary Table 3 Medium Office prototype building model specifications.

**Items**	**Specifications**
Total Floor Area (m^2)	4982.19
Number of Floors	3
Window-to-Wall Ratio	33%
Window Locations	Evenly distributed along four façades
Floor to floor height (m)	3.96
Floor to ceiling height (m)	2.74
Glazing sill height (m)	1.02

Supplementary Table 4 Climate zones of various cities around the world.

**Cities**	**Climate zones[21]**
Singapore, Singapore	Af, Am, Aw
Honolulu, America	BSh, BWh
Turpan, China	BSk, BWk
Tokyo, Japan	Csa, Cwa, Cfa
London, Britain	Csb, Cwb, Cfb
Reykjavik, Iceland	Csc, Cwc, Cfc
Beijing, China	Dsa, Dwa, Dfa
Oslo, Norway	Dsb, Dwb, Dfb
Anchorage, America	Dsc, Dwc, Dfc
Yakutsk, Russia	Dsd, Dwd, Dfd, ET, EF

References

1. Mayer, K. M. & Hafner, J. H. Localized Surface Plasmon Resonance Sensors. *Chemical Reviews* **111**, 3828-3857 (2011).

2. Luther, J. M., Jain, P. K., Ewers, T. & Alivisatos, A. P. Localized surface plasmon resonances arising from free carriers in doped quantum dots. *Nature Materials* **10**, 361-366 (2011).

3. Garcia, G. et al. Dynamically modulating the surface plasmon resonance of doped semiconductor nanocrystals. *Nano Letters* **11**, 4415-4420 (2011).

4. Garcia, G. et al. Near-Infrared Spectrally Selective Plasmonic Electrochromic Thin Films. *Advanced Optical Materials* **1**, 215-220 (2013).

5. Demiryont, H. & Moorehead, D. Electrochromic emissivity modulator for spacecraft thermal management. *Solar Energy Materials and Solar Cells* **93**, 2075-2078 (2009).

6. Sauvet, K., Sauques, L. & Rougier, A. IR electrochromic WO_3 thin films: From optimization to devices. *Solar Energy Materials and Solar Cells* **93**, 2045-2049 (2009).

7. Mandal, J. et al. $Li_4Ti_5O_{12}$: A Visible-to-Infrared Broadband Electrochromic Material for Optical and Thermal Management. *Advanced Functional Materials* **28**, 1802180 (2018).

8. Li, M., Liu, D., Cheng, H., Peng, L. & Zu, M. Manipulating metals for adaptive thermal camouflage. *Science Advances* **6**, eaba3494 (2020).

9. Rao, Y. et al. Ultra-Wideband Transparent Conductive Electrode for Electrochromic Synergistic Solar and Radiative Heat Management. *ACS Energy Letters* **6**, 3906-3915

(2021).

10. Qu, Y. et al. Thermal camouflage based on the phase-changing material GST. *Light: Science & Applications* **7**, 1-10 (2018).

11. Morin, S. A. et al. Camouflage and Display for Soft Machines. *Science* **337**, 828-832 (2012).

12. Agrawal, A. et al. Rationalizing the Impact of Surface Depletion on Electrochemical Modulation of Plasmon Resonance Absorption in Metal Oxide Nanocrystals. *ACS Photonics* **5**, 2044-2050 (2018).

13. Staller, C. M. et al. Tuning Nanocrystal Surface Depletion by Controlling Dopant Distribution as a Route Toward Enhanced Film Conductivity. *Nano Letters* **18**, 2870-2878 (2018).

14. Zandi, O. et al. Impacts of surface depletion on the plasmonic properties of doped semiconductor nanocrystals. *Nature Materials* **17**, 710-717 (2018).

15. Seiwatz, R. & Green, M. Space Charge Calculations for Semiconductors. *Journal of Applied Physics* **29**, 1034-1040 (1958).

16. Jia, J., Takasaki, A., Oka, N. & Shigesato, Y. Experimental observation on the Fermi level shift in polycrystalline Al-doped ZnO films. *Journal of Applied Physics* **112**, (2012).

17. Sernelius, B. E., Berggren, K., Jin, Z., Hamberg, I. I. & Granqvist, C. G. Band-gap tailoring of ZnO by means of heavy Al doping. *Physical Review B* **37**, 10244-10248 (1988).

18. Ling, L., Zhu, R., Gu, Y. & Chen, Z. Doped semiconductor nanoparticles for possible daytime radiative cooling applications. *Semiconductor Science and Technology* **35**, 075018 (2020).

19. Deru, M. et al. *U.S. Department of Energy Commercial Reference Building Models of the National Building Stock* (National Renewable Energy Laboratory, 2011).

20. Goel et al. Enhancements to ASHRAE Standard 90.1 Prototype Building Models. Pacific Northwest National Lab.(PNNL), Richland, WA (United States) https://doi.org/10.2172/1764628 (2014).

21. Beck, H. E. et al. Present and future Köppen-Geiger climate classification maps at 1-km resolution. *Scientific Data* **5**, 180214 (2018).

22. Bruckner, T. et al. *Technology-specific cost and performance parameters [annex III].Climate Change 2014: Mitigation of Climate Change* (Cambridge University Press, 2014).

23. Kim, H. et al. VO_2-based switchable radiator for spacecraft thermal control. *Scientific Reports* **9**, 11329 (2019).

24. Osiander, R., Firebaugh, S. L., Champion, J. L., Farrar, D. & Darrin, M. A. G. Microelectromechanical devices for satellite thermal control. *IEEE Sensors Journal* **4**, 525-531 (2004).

25. Amore, L. J. et al. Optimized RF-transparent antenna sunshield membrane. US5373306A, (1994).

26. Chandrasekhar, P. et al. Large, Switchable Electrochromism in the Visible Through

Far-Infrared in Conducting Polymer Devices. *Advanced Functional Materials* **12**, 95-103 (2002).

27. Ergoktas, M. S. et al. Multispectral graphene-based electro-optical surfaces with reversible tunability from visible to microwave wavelengths. *Nature Photonics* **15**, 493-498 (2021).

28. Sun, Y. et al. Large-Scale Multifunctional Carbon Nanotube Thin Film as Effective Mid-Infrared Radiation Modulator with Long-Term Stability. *Advanced Optical Materials* **9**, 2001216 (2020).

29. Xu, C., Stiubianu, G. T. & Gorodetsky, A. A. Adaptive infrared-reflecting systems inspired by cephalopods. *Science* **359**, 1495-1500 (2018).

30. Leung, E. M. et al. A dynamic thermoregulatory material inspired by squid skin. *Nature Communications* **10**, 1-10 (2019).

31. Krishna, A. *et al.* Ultraviolet to Mid-Infrared Emissivity Control by Mechanically Reconfigurable Graphene. *Nano Letters* **19**, 5086-5092 (2019).

32. Qu, Y. et al. Dynamic Thermal Emission Control Based on Ultrathin Plasmonic Metamaterials Including Phase-Changing Material GST. *Laser & Photonics Reviews* **11**, 1700091 (2017).

33. Xiao, L. et al. Fast Adaptive Thermal Camouflage Based on Flexible VO_2/Graphene/CNT Thin Films. *Nano Letters* **15**, 8365-8370 (2015).

34. Sun, K. et al. VO_2 metasurface smart thermal emitter with high visual transparency for passive radiative cooling regulation in space and terrestrial applications.

Nanophotonics **11**, 4101-4114 (2022).

35. Wang, S. et al. Scalable thermochromic smart windows with passive radiative cooling regulation. *Science* **374**, 1501-1504 (2021).

36. Tang, K. et al. Temperature-adaptive radiative coating for all-season household thermal regulation. *Science* **374**, 1504-1509 (2021).

37. Tian, Y. et al. A comprehensive study of electrochromic device with variable infrared emissivity based on polyaniline conducting polymer. *Solar Energy Materials and Solar Cells* **170**, 120-126 (2017).

38. Sui, C. et al. Aqueous mid-infrared electrically switchable opaque building envelopes for all-season radiative thermoregulation. Preprint at https://10.26434/chemrxiv-2022-vldj6 (2022).

39. Inoue, T., Zoysa, M. D., Asano, T. & Noda, S. Realization of dynamic thermal emission control. *Nature Materials* **13**, 928-931 (2014).

40. Brar, V. W. et al. Electronic modulation of infrared radiation in graphene plasmonic resonators. *Nature Communications* **6**, 7032, (2015).
