# Supplementary material for: Transparent dynamic infrared emissivity regulators
Source: Nat Commun. 2023 Aug 22;14:5087. doi: 10.1038/s41467-023-40902-w (PMC10444874; doi:10.1038/s41467-023-40902-w)
Supplement: Supplementary file 3 — Description of Additional Supplementary Files [file 41467_2023_40902_MOESM3_ESM.pdf]

Description of Additional Supplementary Files

File Name: Supplementary Movie 1
Description: Real-time visible and infrared thermal movie of TDIE regulators, recorded by a Nikon D3100 digital camera and FLIR T1050sc infrared camera.

File Name: Supplementary Movie 2
Description: Real-time visible and infrared thermal movie of SES roofs and SES windows, showing different mode in Supplementary Fig. 41 and Fig. 42.

File Name: Supplementary Movie 3
Description: Real time visible and infrared thermal movie of multispectral display.
